# Biochar supported metallo-inorganic nanocomposite: A green approach for decontamination of heavy metals from water

**DOI:** 10.1371/journal.pone.0289069

**Published:** 2023-09-14

**Authors:** Sana Khalid, Muhammad Nawaz Chaudhary, Rabia Nazir, Sajid Rashid Ahmad, Naqi Hussain, Yaseen Ayub, Muhammad Ibrar

**Affiliations:** 1 College of Earth & Environmental Sciences, University of the Punjab, Lahore, Pakistan; 2 Department of Environmental Sciences & Policy, Lahore School of Economics (LSE), Lahore, Pakistan; 3 Pakistan Council of Scientific and Industrial Research Laboratories Complex, Lahore, Pakistan; 4 Department of Chemistry, Forman Christian College, Lahore, Punjab, Pakistan; 5 Department of Chemistry, Lahore Garrison University, Lahore, Pakistan; King Saud University, SAUDI ARABIA

## Abstract

Heavy metal contamination of water has become a global environmental burden, which has stirred up agitation worldwide. Fabrication of adsorbents utilizing either low cost, environment friendly materials or waste products can be helpful in remediating environmental pollution. The current study evolved around the synthesis of nanocomposites derived from such raw precursors like spent tea waste biochar, hydroxyapatite, and clays. In this context, two nanocomposites, namely manganese ferrite doped hydroxyapatite/kaolinite/biochar (TK-NC) and manganese ferrite doped hydroxyapatite/vermiculite/biochar (TV-NC), were synthesized followed by their employment for decontamination of heavy metals from aqueous media. TK-NC and TV-NC exhibited the crystallite sizes in the range of 2.55–5.94 nm as obtained by Debye Scherrer Equation and Williamsons–Hall equation The fabricated nanocomposites were characterized using FT-IR, SEM-EDX, and powder XRD. Batch adsorption studies were performed, and influence of different adsorption parameters (contact time, reaction temperature, solution pH, adsorbent dose, and initial adsorbate concentration) on metal adsorption was examined. Thermodynamic studies revealed that the adsorption of Cr(VI), Ni(II) and Cu(II) on TK-NC and TV-NC was endothermic (+*ΔH°*) and indicates disorderness (+*ΔS*°) at the solid-liquid interface owing to the strong affinity of metal ions with adsorbent. The heavy metal uptake selectivity followed the following decreasing order; Cr(VI) > Cu(II) > Ni(II) by both nanocomposites, with adsorption capacities falling in the range of 204.68–343.05 mg g^-1^. Several adsorption kinetic and isotherm models were applied to experimentally calculated data, which suggest favorable adsorption of Cr(VI), Ni(II) and Cu(II) by TK-NC and TV-NC from the system while obeying general-order kinetics and R-P adsorption model, conferring the transition in adsorption kinetics order and involvement of multiple adsorption process.

## 1. Introduction

The global shortage of freshwater resources, coupled with incessant accelerating human demands for safe and clean water, poor water management practices, and inequitable sharing of water, has urged the requisite to preserve current water resources for socioeconomic growth and environmental sustainability [1]. The modern human civilization and industrialization have resulted in indiscriminate discharge of bulk volumes of effluents loaded with multiple toxic pollutants, including heavy metals, chlorides, sulfates, nitrates, dyes, pesticides, pharmaceuticals, phenols, microbes, etc., into aquatic environments, leading to colossal environmental impairment [2]. Heavy metal contamination of water has become a global environmental challenge that causes massive environmental destruction and potential health damages to all life forms owing to their toxicity, persistence, non-biodegradability, and bio-accumulation once they entered the food chain [3]. Copper (Cu(II)), chromium (Cr(VI)) and nickel (Ni(II)) all are classified as highly toxic heavy metals even in trace quantities and have widespread applications in numerous industrial sectors, including tanneries, metal electroplating, paper, paints, textile dyeing, fertilizers, pesticides, automotive electronic equipment, etc. These heavy metals’ toxicity has been found to be linked with incidence of heart, liver, lungs, kidneys, and skin disease, neuropsychological disorders, genetic alterations, cancer, etc. [3], which has urged the need for their effective decontamination from environment.

Removal of contaminants, particularly heavy metals, from wastewater is an extremely challenging task. A multitude of water purification technologies, including chemical precipitation, coagulation and flocculation, ion exchange, ozonation, membrane filtration, electro-dialysis, biological treatment, and adsorption, have been tested for abatement of these pollutants from aqueous solutions [4]. Though adsorption is considered a highly compelling method for wastewater decontamination owing to its convenient, efficient, and environment friendly nature. The immense attention has been given on fabricating economically viable and environment friendly adsorbents, particularly those that are derived either from natural precursors and waste materials or the composite of these. Such adsorbents deliver the dual purpose of waste management and environmental remediation in addition to addressing the green-chemistry principles. In this perspective, several adsorbents have been fabricated which exhibit effective remediation potential for heavy metals, anions, organic pollutants, and microbes [5–7]. The materials like Coal fly ash modified hydroxyapatite, iron doped Ethiopian nano clay, Hydrocalcite/hydroxyapatite impregnated carbon nanotubes, magnetic nanoparticles modified chitosan incorporated biochar, iron nanoparticles impregnated tea waste, magnetic nanoparticles decorated tea waste biochar, etc. have been employed for removal of multiple heavy metals (Cu(II), Ni(II), Cr(VI)) with uptake capacities ranging from 11.97–200.80 mg g^-1^, but consumed prolonged time (2–72 hours) to reach equilibrium stage [8–13] (S1 Table). Among these multifarious adsorbents, biochar-based ones have attracted much more attention owing to their low cost and easy availability of the raw materials especially if they are waste-derived. Based on the source and method of preparation, the adsorption capacities of these biochars vary from one metal to another. Pristine biochar was also reported to have low sorption potential, and hence various modifications are also suggested for improving metal uptake. These modifications include doping with inorganic, organic, or a combination of both to impact the adsorption capacities [14]. In this regard, several biochar are used, as highlighted in S1 Table, which require excessive time to result in an appreciable adsorption capacity. Hence, there is a need to design a modified biochar that can amicably reduce the time to remove multiple contaminants from the wastewater while achieving high adsorption capacities. Therefore, the current study offers the fabrication of a novel biochar-based adsorbent that can overcome this issue in addition to meeting the following objectives: 1) repurposing of natural feedstock into plausibly efficient adsorbent; 2) employment of fabricated material for abatement of target heavy metals while achieving profound adsorption potential in reduced time.

## 2. Experimental section

### 2.1. Materials and methods

All the chemical reagents used in this study were of analytical-grade purity. The solutions were prepared in distilled water. Manganese sulphate monohydrate, ferrous sulphate heptahydrate, potassium dichromate, sodium hydroxide, and nitric acid were purchased from Sigma Aldrich, and anhydrous calcium chloride, anhydrous disodium hydrogen phosphate, copper sulphate pentahydrate, and nickel chloride hexahydrate were obtained from Daejung Chemicals. Hydrochloric acid was procured from Pakistan Council of Scientific and Industrial Research (PCSIR) Lahore, Pakistan. Kaolinite and vermiculite clays were purchased from a local vendor in Akbari market, Lahore, Pakistan. All the chemicals were used as received without further purification.

### 2.2. Synthesis of nanocomposites

#### 2.2.1. Synthesis of spent tea waste biochar (TBC)

Spent black tea biochar was prepared by method described by Butt *et al*. [13] with little modifications. For this, spent black tea waste was collected from a local cafeteria located in Lahore, Pakistan. Spent tea waste underwent several cycles of washing with hot boiling distilled for removing color and soluble impurities, followed by its drying in oven at 80°C overnight. The dried tea waste was pulverized into fine powder and passed through 250micron sieve, followed by its treatment with 2M HCl in 1:2 w/v ratio at 80°C for 2 hours under constant agitation. The resultant solution mixture was let to cool down to room temperature, filtered, and tea waste residue was washed with distilled water until neutralized. Subsequently, acid activated spent tea waste was pyrolyzed at 500°C for 2 hours in oxygen deficient environment to yield biochar. For this purpose, the continuous flow of nitrogen was maintained in the tube furnace that was locally manufactured in PCSIR.

#### 2.2.2. Synthesis of hydroxyapatite nanoparticles (HA)

HA nanoparticles were synthesized by chemical precipitation method described by Le *et al*. [15] with little modifications. For this, to the 7.6mM anhydrous Na_2_HPO_4_ (0.54g/500ml) solution, 13mM anhydrous CaCl_2_ (0.72g/500ml) solution was added dropwise while keeping temperature at 65⁰C under vigorous stirring. During the reaction, pH of the solution was maintained at 10.5 by using 0.1M NaOH. After completion of reaction, the contents of reaction flask were allowed to rest overnight for aging of precipitates. Subsequently, the precipitates were filtered, washed with distilled water, and oven dried at 110°C overnight.

#### 2.2.3. Synthesis of manganese ferrite doped hydroxyapatite/clay/biochar nanocomposites (TK-NC and TV-NC)

In this study, two types of clays (i.e., kaolinite (KC) and vermiculite (VC)) were alternatively assimilated with activated tea waste biochar (TBC), calcium hydroxyapatite (HA), and transition metals precursors (manganese sulphate and ferrous sulphate) to fabricate manganese ferrite doped hydroxyapatite/kaolinite/biochar (TK-NC) and manganese ferrite doped hydroxyapatite/vermiculite/biochar (TV-NC) nanocomposites.

**Fig 1:**
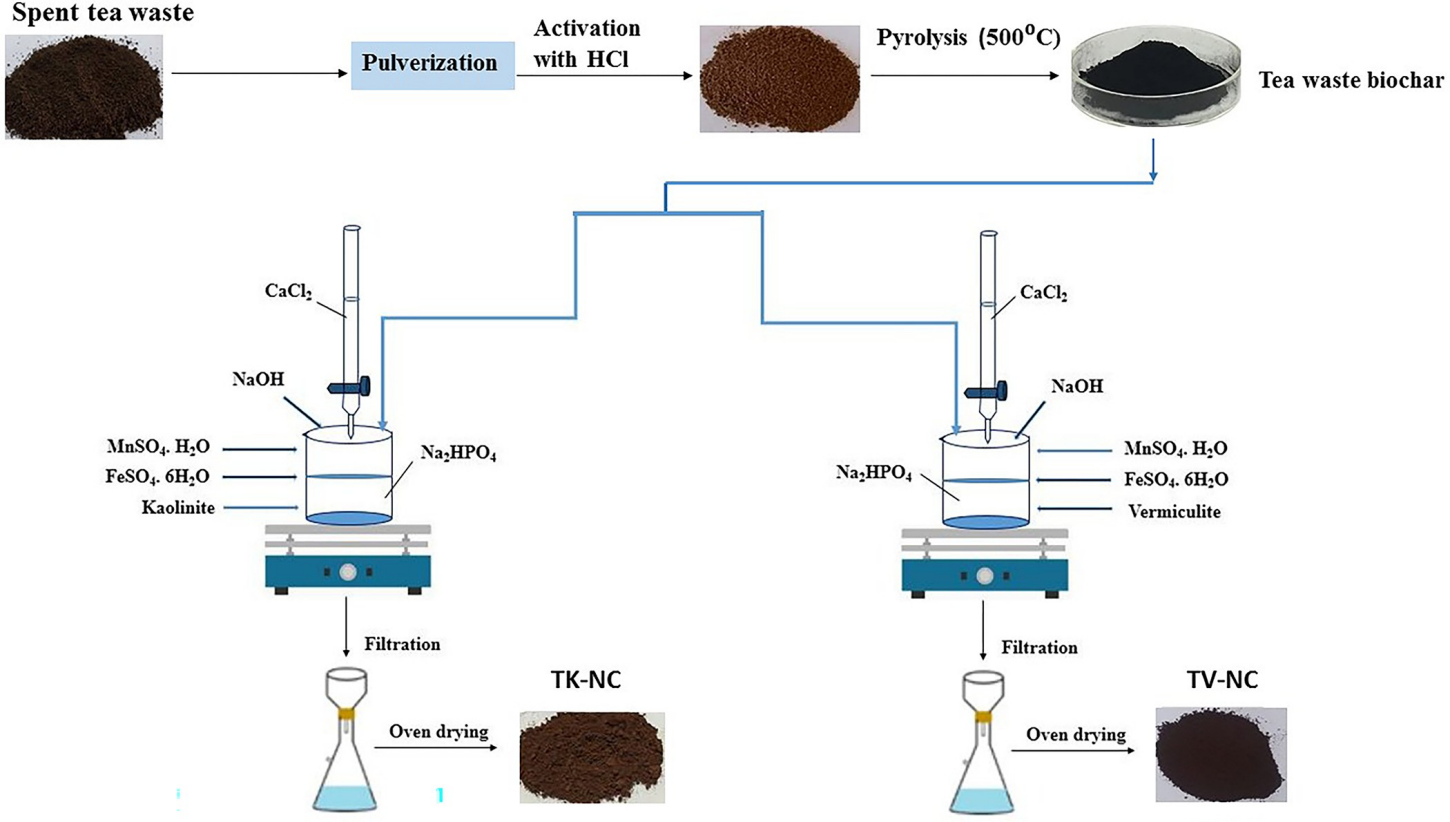
Flowsheet diagram of synthesis of TK-NC and TV-NC nanocomposites.

Both nanocomposites were fabricated by method described by Wang *et al*. [16] with modifications. For this, TBC (0.3 g) and clay (KC/VC, 0.3 g) were simultaneously introduced into 2 L conical flask containing 7.6mM anhydrous Na_2_HPO_4_ solution (500ml) at 65°C under constant agitation. After half an hour, 1.8mM FeSO_4_.7H_2_O and 0.9mM MnSO_4_.H_2_O were added into the flask, and the reaction mixture was vigorously stirred for 30 minutes for homogenous mixing. To this suspension, 13mM CaCl_2_ solution (500ml) was added dropwise while maintaining pH of reaction 10.5 using 0.1M NaOH solution. After completion of reaction, the contents in the reaction flask were stand overnight for aging. Subsequently, the resulting greyish-brown precipitates were filtered, washed with distilled water, and dried in oven at 110°C. The Fig 1 presents the schematic illustration of stepwise fabrication of TK-NC and TV-NC.

### 2.3. Characterization of nanocomposites

Characterization of fabricated nanocomposites (TK-NC and TV-NC) and their pristine components was carried out by using different techniques. Scanning electron microscopy and energy dispersive X-ray (SEM-EDX) analysis were done using Nova NanoSEM 450 field-emission scanning electron microscope (FE-SEM) for examining the surface morphology and chemical composition of fabricated biochar supported metallo-inorganic nanocomposites and their pristine components. Fourier transform infrared spectroscopy (FTIR) was studied over a range 4000–600 cm^-1^ by using Tensor-27 performed to determine the surface functional groups of raw counterparts and fabricated nanocomposites. The crystalline nature and phase of samples were investigated by using X-ray Diffractometer (Bruker, D2-Phaser). Zeta potential of nanocomposites was determined by using the Malvern Zetasizer Nano ZSP.

### 2.4. Methylene blue value (MBV)

MBV was estimated to screen out potentially efficient adsorbents for metal ions adsorption studies. MBV is defined as the value of methylene blue standard solution in (ml) that is decolorized with 0.1 g of sample used. The test was performed using the standard protocol described by Cefic (1986) [17].

To estimate the MBV, 5 mg L^-1^ methylene blue solution in diluted acetic acid (0.25%, v/v) was prepared from methylene blue aqueous stock solution (1000 mg L^-1^), and its absorbance was recorded at 620 nm (0.84 ± 0.01). 0.1 g of fabricated nanocomposites and their pristine counterparts were contacted with 5 ml of methylene blue experimental solution at room temperature under constant stirring. The samples were shaken until discoloration was observed, which was done by spreading few drops of treated methylene blue solution on a white surface and observing its hue. Then, another 1 ml of methylene blue solution was added and stirred to discolor. The inclusion of methylene blue experimental solution in 1 ml portions was repeated as long as discoloration occurred as noticed by a clear or almost colorless film on the surface. After appearance of color in treated sample, the methylene blue solution was added to the reaction vessel until the same absorbance was achieved as of the untreated methylene blue solution. The volume (ml) of methylene blue solution decolorized until the endpoint achieved was noted and recorded as MBV.

### 2.5. Adsorption studies

Batch experiments were performed to investigate the adsorption of target metal ions (Cr(VI), Ni(II) and Cu(II)) onto synthesized nanocomposites under varying conditions of different experimental parameters (i.e., contact time, temperature, solution pH, adsorbent dose, and initial metal ion concentration). To perform this aqueous stock solution (1000 mg L^-1^) of Cr(VI), Cu(II) and Ni(II) were separately prepared from potassium dichromate (K_2_Cr_2_O_7_), copper sulphate pentahydrate (CuSO_4_. 5H_2_O), and nickel chloride hexahydrate (NiCl_2_. 6H_2_O), respectively. The working solutions of preferred concentrations of respective metals were made by diluting the stock solutions.

For a series of experiments to be carried out to optimize the process parameters required for maximum metal adsorption, 50 ml of known concentration of respective metal ion solution (300–1000 mg L^-1^) taken in a conical flask with a known dose of adsorbent (0.1–0.5 g) was stirred at 200 rpm for a given time period (2–90 min) and temperature (30–80°C). The pH (1.0–8.0) of the solution was adjusted with the help of 0.1M HCl and 0.1 M NaOH. Afterwards, the sample suspension was filtered using cannula filtration to separate out solid and liquid phases. The residual amount of metal in filtrate was analyzed with the help of Flame atomic absorption spectrometer (PerkinElmer AAnalyst 200). The percentage removal efficiency (RE%) and the concentration of adsorbate sorbed on adsorbent (adsorption capacity (mg g^-1^), q_exp_) were calculated using Eqs (1) and (2).

$$RE(\%)=[\frac{C_{i}-C_{f}}{C_{i}}]\times 100$$ (1)

$$q_{exp}=(C_{i}-C_{f})\times \frac{V}{W}$$ (2)

where, *C*_*i*_ (mg L^-1^) and *C*_*f*_ (mg L^-1^) are the initial and final concentrations of adsorbate in solution, respectively; V (L) is the volume of solution; and W (g) is the mass of adsorbent.

## 3. Results and discussion

### 3.1. Characterization of nanocomposites and their pristine components

#### 3.1.1. Hydroxyapatite (HA)

**Fig 2:**
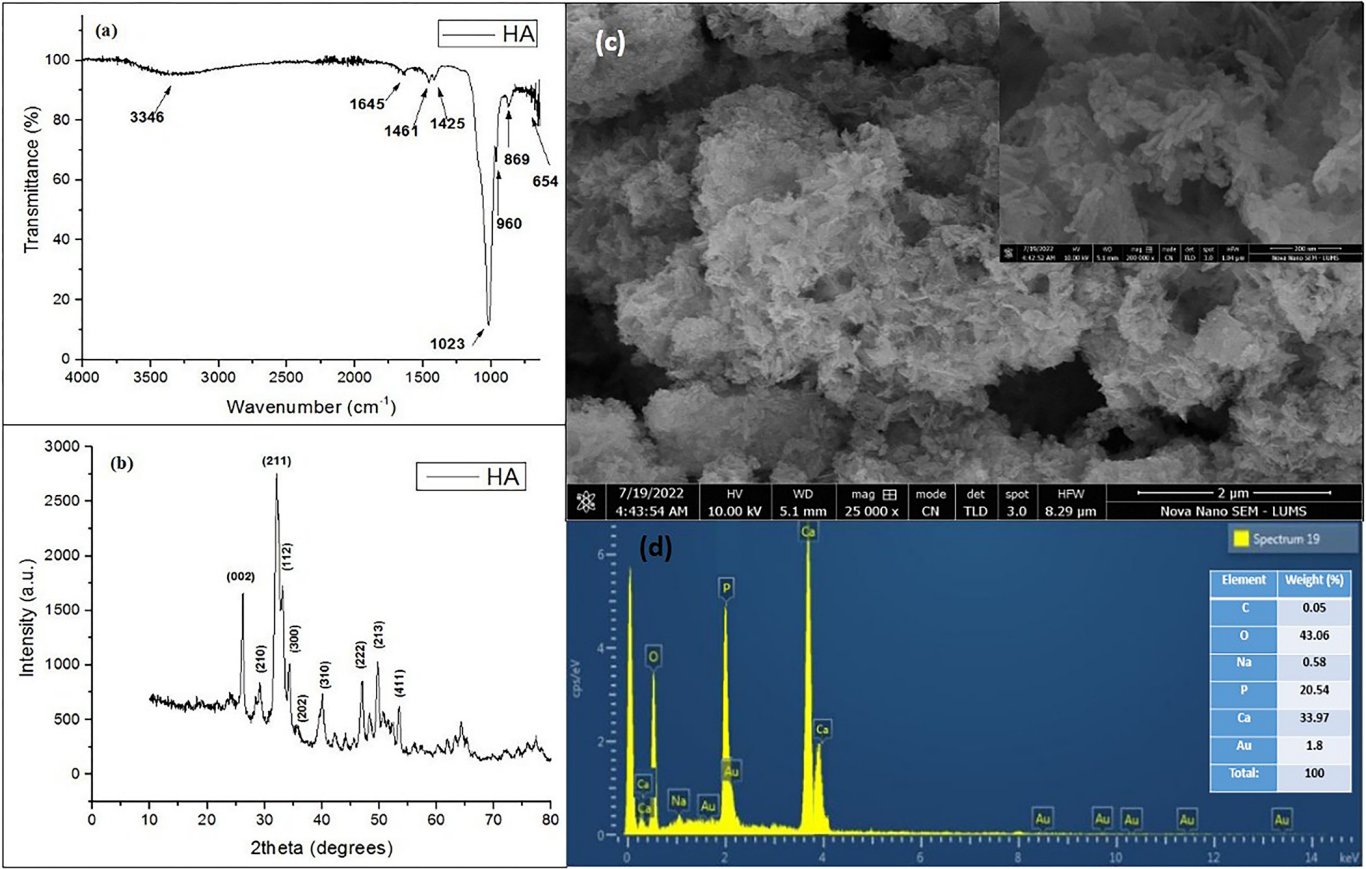
a) FTIR spectrum, b) powder XRD diffractogram, c) SEM micrograph with high magnification image in inset and d) EDX spectra with elemental composition in inset of HA.

The FTIR spectrum of HA is presented in Fig 2A. The weak signals observed at 3364 cm^−1^ and 1645 cm^−1^ are ascribed to stretching vibrations of–OH group due to adsorbed water molecules in HA lattice [18]. The peaks evidenced at 1461 and 1425 cm^−1^ correspond to CO_3_^2-^ group. The band appearing at 1023 cm^−1^ is related to asymmetric bending mode of PO_4_^3-^ while the peak at 960 cm^−1^ represents the symmetric stretching of PO_4_^3-^ group [19]. The signals at 869 cm^−1^ is due to P-OH corresponding to HPO_4_^-^, which is the characteristic feature of hydroxyapatite [19].

Fig 2B represents powder XRD diffractogram of HA. The peak planes referring to hkl values of (002), (210), (211), (112), (300), (202), (310), (222), (213) and (411) are indexed to calcium hydroxyapatite, which are in good agreement with JCPDS card no. 09–0432 [20]. In addition to that, XRD data is used to calculate average crystallite size by using D-Scherrer (DS) equation i.e., Eq 3 [21].


D=Kλβcosθ (3)

While D refers to crystallite size in nm, λ = 0.154 nm for Cu Ҡα, X-ray radiation, shape factor is k = 0.93, peak position (θ) and β is known as full width at half maximum (FWHM). The average crystallite size obtained for HA is 5.64 nm. The Williamsons–Hall equation (WH) as given in Eq 4 was also utilized to measure the average size of crystallite of all types of nanocomposites, which were more suitable due to widening of peaks because of crystallite size and lattice strain [22].


D=kλβcosθ+4εsinθ (4)

βcosθ=kλD+4εsinθ (5)

**Fig 3:**
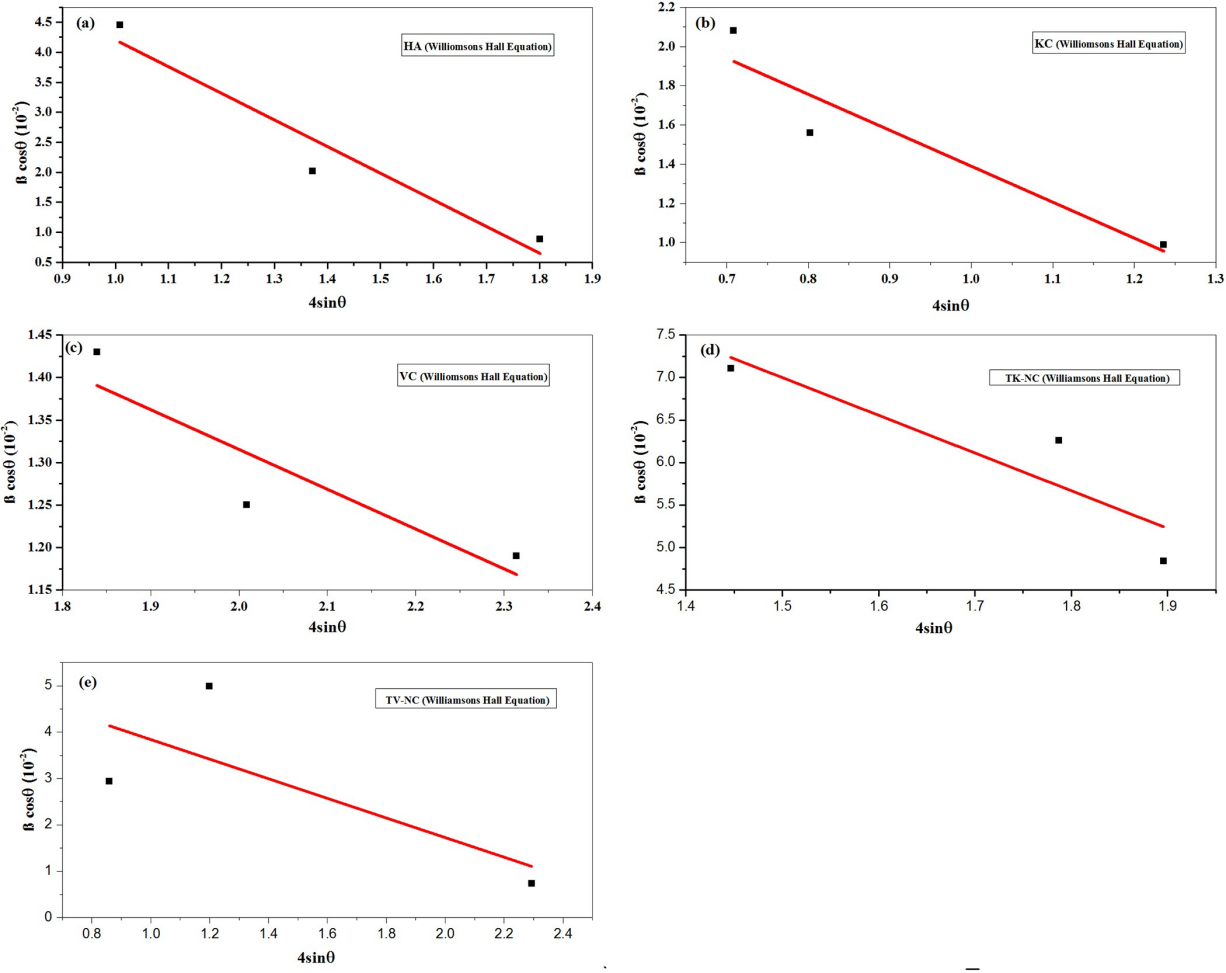
Calculation of slop and intercepts by liner plot of Williamsons Hall equation (βcosθ vs 4sinθ) for (a) HA, (b) KC, (c) VC, (d) TK-NC, (e) TV-NC.

Where, crystallite size (D) and lattice strain (ϵ) were measured through intercept and slope by linear plot between βcosθ vs 4sin θ by the Eq (5) which is manipulated by Eq (4) (Fig 3). The crystallize sizes as obtained from DS and WH equations (Eqs 3–5; Fig 3) comes out to be 11.26 and 3.43nm, respectively.

The SEM micrograph of HA nanoparticles (Fig 2C) depicts a rod-like structure of HA with a spongy texture and appearing in dense aggregates forming micro clusters together. These findings are in accordance with previous studies [23].

The EDX analysis (Fig 2D) confirmed the presence of Ca, P and O with elemental weight % of 33.97, 20.54, and 43.06, respectively, which are the chief constituents of HA. The Ca/P ratio as calculated using EDX quantitative data came out to be 1.65, which conforms to the stoichiometric molar ratio (1.67) of hydroxyapatite [18]. A trace amount of C (0.05%) occurs as impurity, while Na (0.58%) is present due to NaOH used for co-precipitation.

#### 3.1.2. Clays

**Fig 4:**
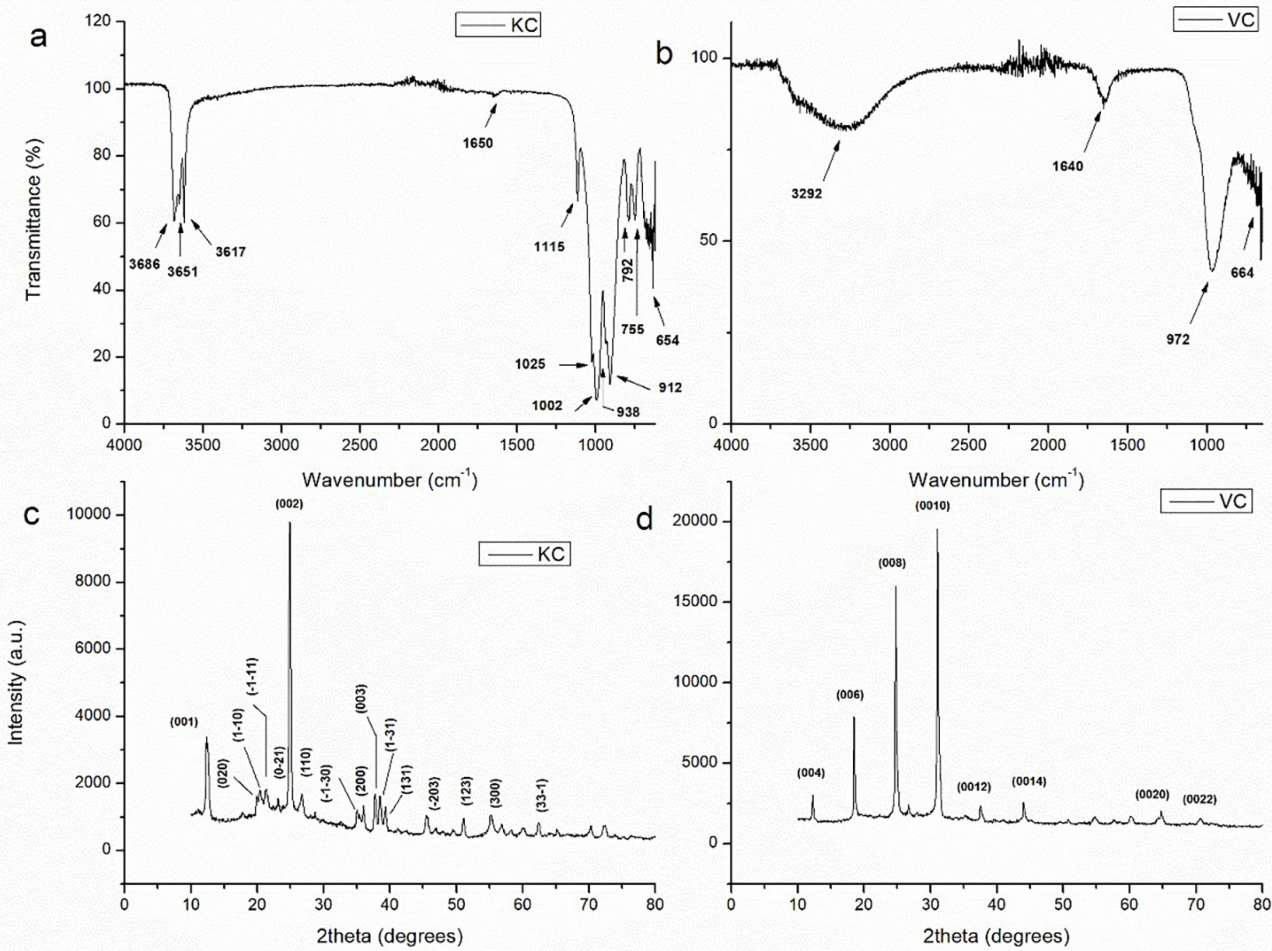
FTIR spectra (a, b) and XRD diffractogram (c, d) of KC and VC, respectively.

The FTIR spectra of procured well-ground clays (kaolinite (KC) and vermiculite (Vc)) are given in Fig 4 which depicts typical peaks of O-H, Si-O and Al-O. In the case of KC (Fig 4A), peaks appearing at 3686, 3651, 3617, and 1650 cm^−1^ are attributed to stretching vibrations of–OH group due to adsorbed water molecules in KC lattice. The appearance of these multi-characteristic bands denotes ordered structure of the KC [24]. The peaks at 1115, 1025, and 1002 cm^−1^ correspond to Si-O group, and the ones appearing at 938 and 912 cm^−1^ are ascribed to Al-OH group [25]. The peaks related to Si-O-Al vibrations are observed at 792 and 755 cm^-1^, while the band at 654 cm^−1^ reflects Al-OH functional group [24]. Similarly, in the case of VC (Fig 4B), the signals observed at 3292 and 1640 cm^−1^ corresponds to stretching and bending vibrations of–OH group, respectively, while the ones appearing at 972 and 664 cm^−1^ attributed to SiO_2_ band and Al-OH stretching, respectively [26, 27].

The XRD diffractogram of KC (Fig 4C) refers to diffraction peaks appearing at 2θ values of 12.48°, 19.8°, 20.4°, 21.3°, 24.98°, 35.1°, and 39.28° corresponding to hkl values of (001), (020), (110), (-1-11), (002), (-1-30), and (1–31), respectively, which are indexed to representative peaks of kaolinite and match well with JCPDS card no. 00-005-0143 [25]. In the case of VC (Fig 3D), the peaks corresponding to lattice planes values of (004), (006), (008), (0010), (0012), (0014), (0020), and (0022) are all related to vermiculite clay, which is in close agreement with JCPDS card no. 16–0613 [28]. The crystallize sizes calculated by using DS and WH equations (Eqs 3–5; Fig 3) for KC and VC are 14.92, 8.38 nm, and 22.9, 32.11 nm, respectively.

**Fig 5:**
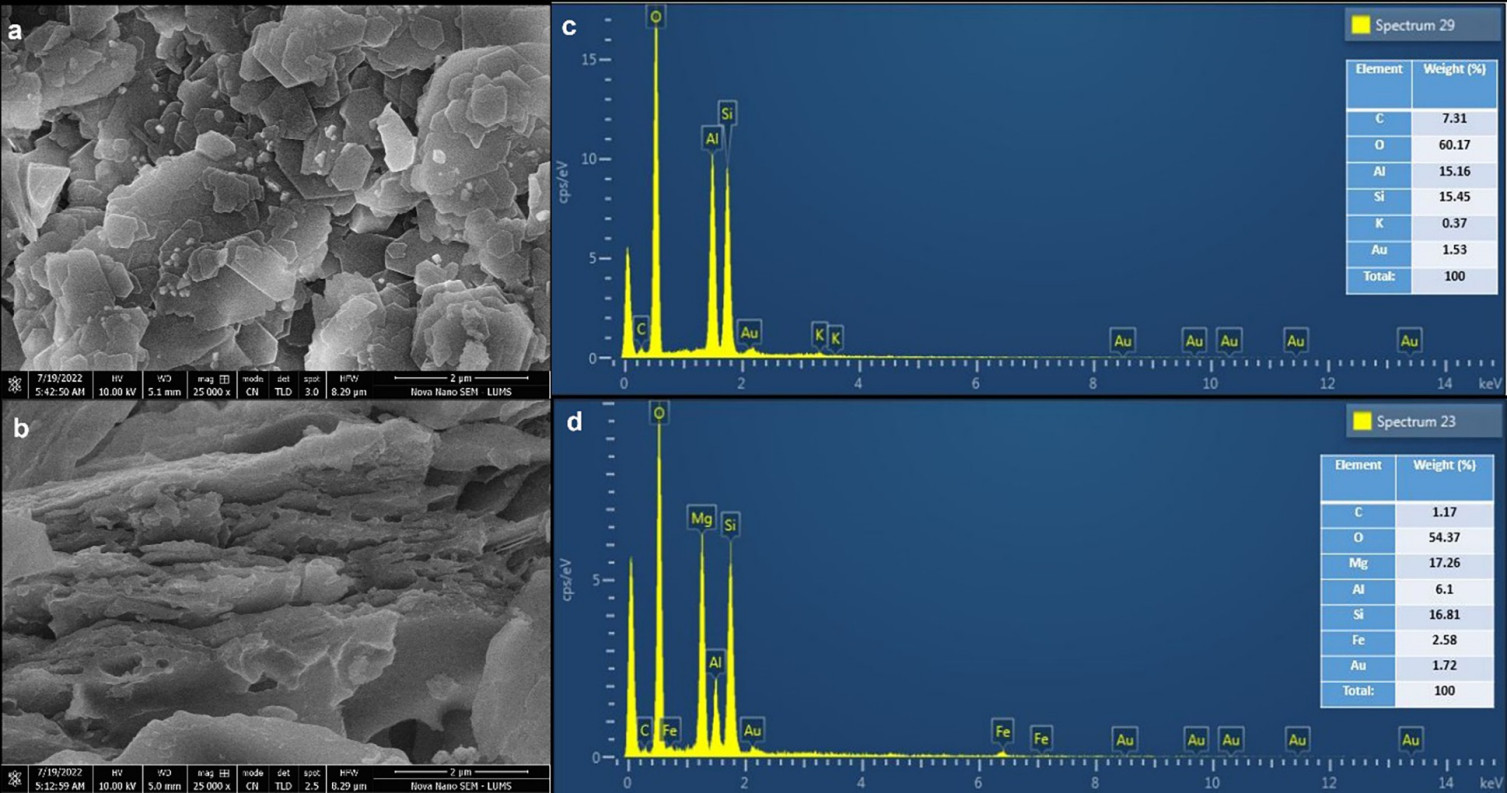
SEM micrograph (a, b) of kaolinite and vermiculite clays along with EDX spectra with elemental composition presented in inset EDX spectra (c, d), respectively.

In addition to that, SEM images of both the clays were also acquired (Fig 5A and 5B), which depict typical multilayer structure. KC clay (Fig 5A) particles exhibiting platelet-like morphology have flat broad planes and thin edges with continuous stacking over one another [25], while the SEM image (Fig 5B) of VC demonstrates multiple lamellar sheeting, presenting a flaky exfoliated architecture with a porous network [29].

EDX spectra of KC and VC along with their elemental composition is presented in Fig 5C and 5D, which illustrates the percentage (%) weight of the major elemental components. In the former case (Fig 5C), O peak is highly conspicuous in the spectrum, with % weight of 60.17%. In addition to that, Al, Si, and K account for 15.16, 15.45, and 0.37%, respectively, with some amount of C (7.31%). The composition of KC is in accordance with the elemental composition of commercial kaolinite reported in literature [30]. Similarly, the EDX of VC (Fig 5D) confirms the presence of O, Mg, Al, Si, Fe, and C with %weight, which is in accordance with the previous studies [31].

#### 3.1.3. Spent tea waste biochar (TBC)

**Fig 6:**
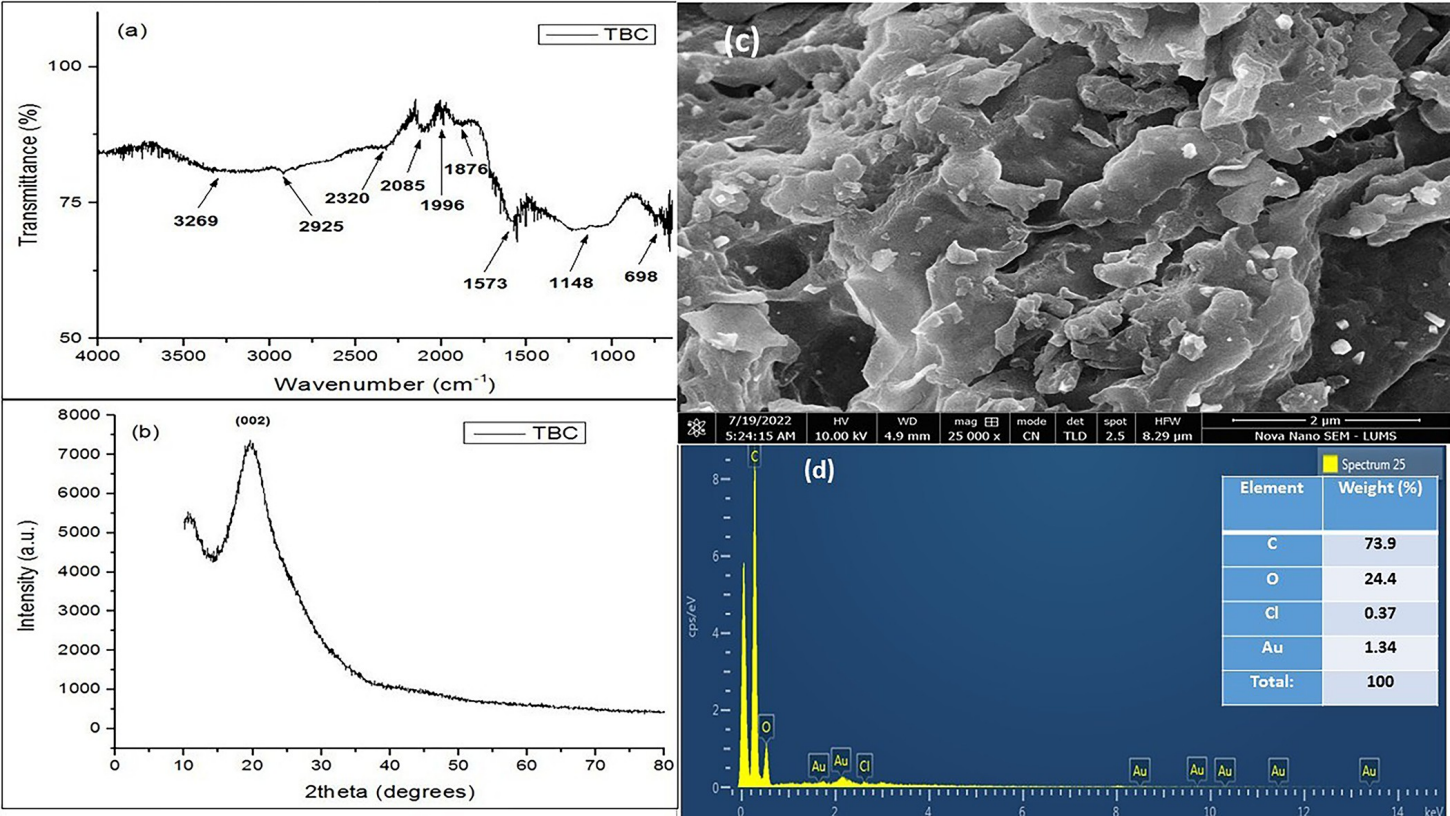
a) FTIR spectrum, b) powder XRD diffractogram, c) SEM micrograph and d) EDX spectrum with elemental composition in inset of TBC.

The FTIR spectrum of TBC is shown in Fig 6A. The broad band (3600–3000 cm^−1^) corresponds to–OH stretching of alcohols and phenolic compounds present in biochar [32]. The peaks at 2925, 2320, and 2085 cm^−1^ are related to C≡C stretching [33]. The signals appearing at 1996 and 1876 cm^−1^ ascribe to C = O group of carboxylic acids, ketones, and lactones [34]. The peaks evidenced at 1573 and 1148 cm^−1^ correspond to stretching vibrations of C = C or C = O and C-O-C groups, respectively [32, 35]. The C-H bending is evidenced at 698 cm^−1^ [34].

The powder XRD diffractogram of TBC (Fig 6B) illustrated a broad peak with hkl plane index (002), which is attributed to C [36]. The broad hump of peak indicates poor crystallinity of TBC, conferring to the amorphous behavior of biochar. The XRD data presented only one peak, which was insufficient for calculating the crystallite size by WH equation. The crystallize size obtained from DS comes out to be 2.51 nm.

SEM image of TBC (Fig 6C) exhibited multilayered porous architecture, comprising of adjacently clumped particles having asymmetrical morphology with rough and uneven edges. The vast number of shards of particulate material attached to the surface of biochar can be clearly seen. EDX spectrum of TBC is presented in Fig 6D, which demonstrates a very conspicuous peak of C, which corresponds to %weight of 73.9%, while the occurrence of O is attributed to oxygen-containing surface functional groups, accounting %weight of 24.4%. Very small quantity of Cl (0.37%) is also present in the biochar owing to the treatment of spent tea waste with hydrochloric acid prior to biochar synthesis.

#### 3.1.4. Nanocomposites (TK-NC and TV-NC)

**Fig 7:**
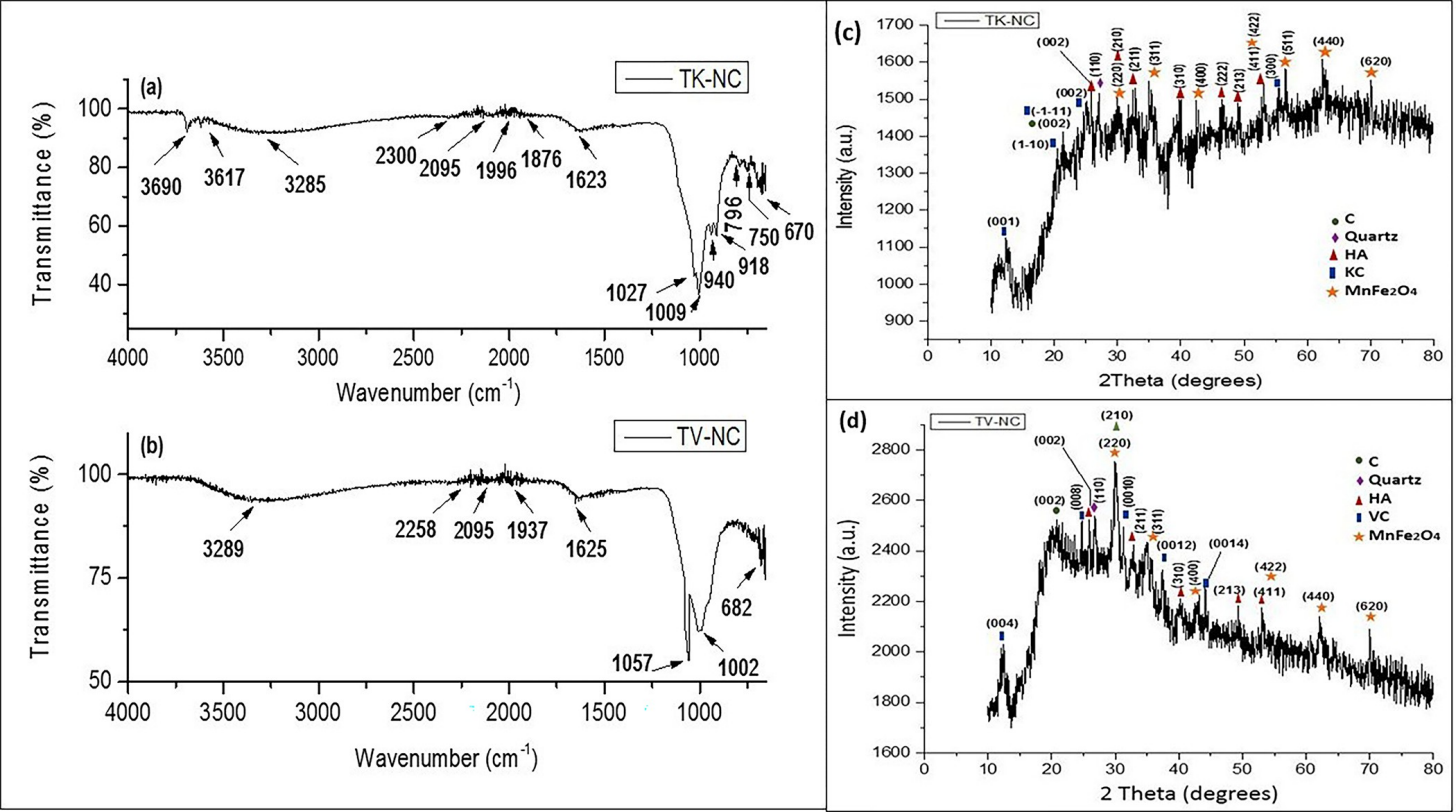
FT-IR (a, b) and powder XRD spectra (c, d) of nanocomposites TK-NC and TV-NC, respectively.

For affirmation of nanocomposites formation, FT-IR, XRD, SEM and EDX analysis were performed. FT-IR spectra of nanocomposites (TK-NC and TV-NC) are presented in Fig 7A, 7B. In the case of TK-NC (Fig 6A), the characteristic peaks of KC, TBC, HA and doped Fe and Mn can be noticed. The well-defined multiple O-H peaks are observed in the range of 3690–3285 cm^−1^ [34]. Similar peaks were noticed in ordered pristine KC (Fig 4A), but in the case of TK-NC (Fig 7A), the peaks are of relatively low intensity with slight peak shifting indicating the formation of composite. In addition to O-H peaks, additional peaks were observed in the TK-NC due to presence of TBC. C≡C stretch peak appearing at 2300 and 2095 cm^−1^ in nanocomposite is evidenced to be little shifted from pristine TBC (i.e., 2320 and 2085 cm^−1^) (Fig 6A), while the signals appearing at 2925 cm^−1^ (C≡C stretching) in TBC spectrum was not appeared in the case of TK-NC. Furthermore, TBC contributed to bands at 1996 and 1876 cm^−1^ ascribing to C = O group of carboxylic acids, ketones and lactones. The peak evidenced at 1623 cm^−1^ corresponds to stretching vibrations of C = C or C = O groups with red shift from 1573 cm^−1^ representing interaction of TBC with other components in the nanocomposite [33, 34]. The appearance of peaks at 1027 and 1009 cm^-1^ correspond to asymmetrical bending vibrations of PO_4_^3-^ of HA and Si-O groups of KC, respectively [19, 24]. The signals at 940 and 918 cm^−1^ are related to Al–OH stretching. The Si-O-Al bond was detected at 796 and 750 cm^−1^, while the peak at 673 cm^−1^ attributed to Al–OH functional group [24, 37].

In the case of TV-NC (Fig 7B), the peak detected at 3289 cm^−1^ is ascribed to O-H stretching vibrations of adsorbed water molecules in the nanocomposite [27, 34]. The shift is recorded for TBC contributed C≡C band resulting in appearing at 2258 and 2095 cm^−1^ [33, 34]. The bands at 1937 and 1625 cm^−1^ accredited the appearance of C = O and C = C groups, respectively. The appearance of PO_4_^3-^ group asymmetrical vibrations at 1057 cm^−1^ affirmed the presence of HA in nanocomposite [19]. The peak appearing at 1002 cm^−1^ corresponds to Si-O group emanating from VC. The Mg/Al-OH vibrations are observed at 682 cm^−1^ [26, 27]. In addition to those multiple peaks presenting in range of 1000–600 cm^-1^ in both nanocomposites are attributed to M-O bond present in Manganese ferrite. The FT-IR analysis of metal-laden TK-NC and TV-NC is also performed to explore the interaction between adsorbent and adsorbate, which is displayed in S1 Fig. The significant changes in band intensity and peak positions of some surface functional groups in both nanocomposites after adsorption of metals are detected as given in S2 Table, suggesting the contribution of corresponding surface functional groups in adsorption of Cu(II), Ni(II) and Cr(VI).

Fig 7C displays the powder XRD diffractogram of TK-NC. The diffraction peak plane hkl (002) is accredited to presence of C from TBC, while the peak observed at hkl (110) corresponds to quartz, which matches well with JCPDS card no. 46–1045 [38, 39]. The peaks at 2θ (12.48°, 20.46°, 21.32°, 24.98°, and 55.6°) corresponding to planes values of (001), (1–10), (-1-11), (002) and (300) are related to KC_,_ which conforms to JCPDS card no. 00-005-0143 [25]. The peak planes corresponding to hkl values of (002), (210), (211), (310), (222), (213), and (411) conform to HA_,_ which matches well with JCPDS card no. 09–0432 [20]. The diffraction peak planes ascribing to hkl values of (220), (311), (400), (422), (511), (440), and (620) are indexed to MnFe_2_O_4_ (JCPDS card no. 10–0319) [40]. The low peak intensity of MnFe_2_O_4_ and hydroxyapatite ascribes to their poor crystalline nature owing to smaller crystallite size suggesting the formation of nanocomposite [33, 41]. XRD of V-NC (Fig 7D) illustrates that the peak plane hkl (002) appearing at 2θ (21.1°) is accredited to C, while the peak at 2θ (26.8°) with hkl value (110) corresponds to quartz from clay, which is in accordance with JCPDS no. 46–1045 [38]. TV-NC exhibited all the characteristic peaks of MnFe_2_O_4_ corresponding to hkl values of (220), (311), (400), (422), (440), and (620), which is in good agreement with JCPDS card no. 10–0319 [40], while lattice planes (004), (008), (0010), and (0012) conform to VC (JCPDS no. 16–0613) [28]. The successful integration of HA in nanocomposite was confirmed by peaks appearing at 2θ (25.8°, 30.1°, 32.8°, 39.9°, 49.4°, 53.1° with hkl values (002), (210), (211), (310), (213) and (411) (JCPDS card no. 09–0432) [20]. The low peak intensity of MnFe_2_O_4_ and hydroxyapatite with peak broadening, in this case as well, indicates poor crystallinity owing to smaller crystallite size suggesting the formation of nanocomposite [33]. The crystallite size determined from DS and WH (Eqs 3–5, Fig 3) comes out to be 5.78 and 2.55 nm for TK-NC and 5.94 and 3.01 nm for TV-NC, conforming the presence of nanocomposites.

**Fig 8:**
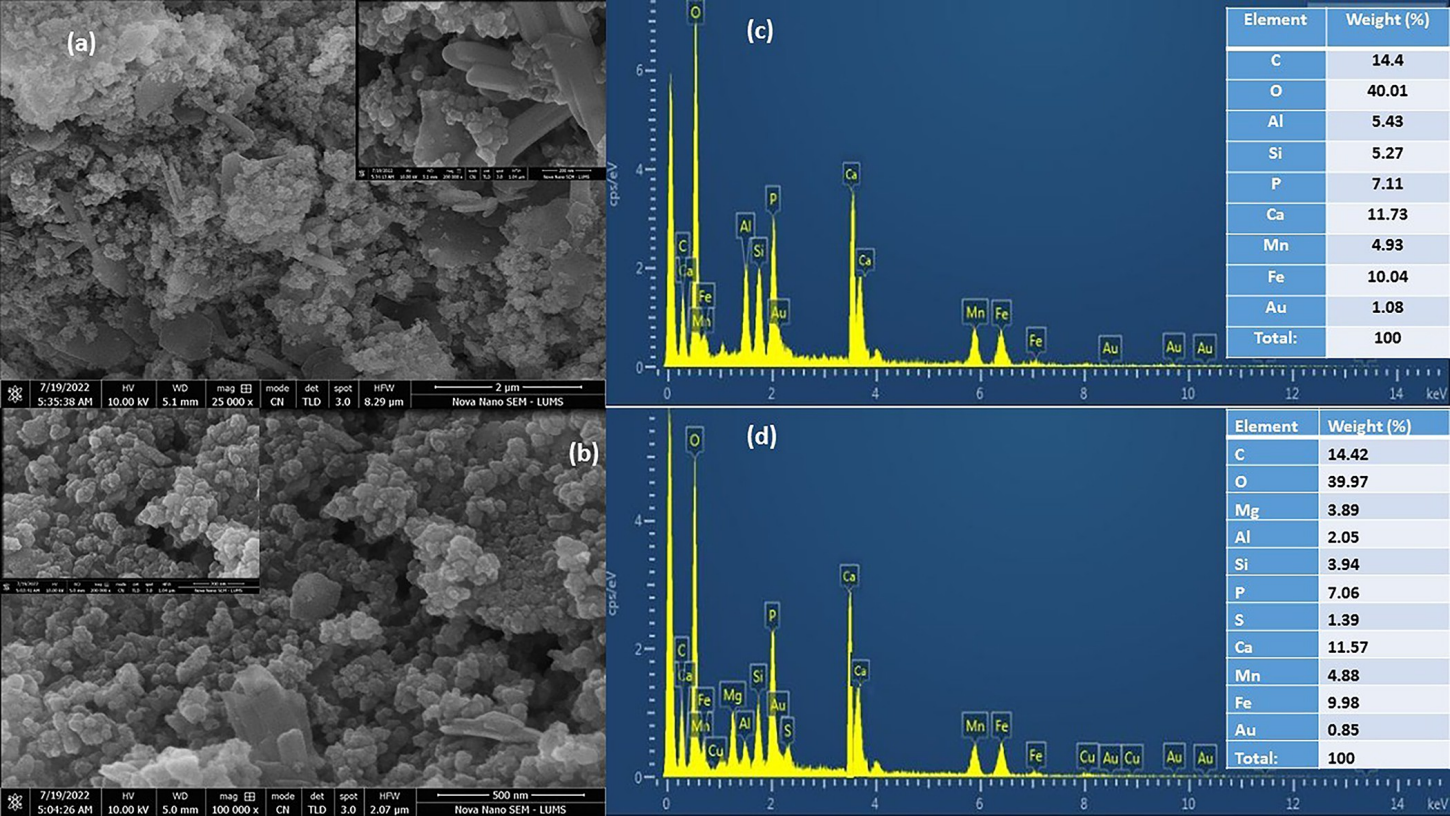
SEM micrographs (a, b) and EDX spectra (c, d) and elemental composition in inset of nanocomposites TK-NC and TV-NC, respectively.

The morphology of the prepared nanocomposites was evaluated by acquiring SEM images (Fig 8). In the case of TK-NC (Fig 8A), KC particles are presented as well-defined structural units dispersed within the nanocomposite framework. The micrograph shows cluster formation of TBC, HA, and MnFe_2_O_4_ nanoparticles over the clay bed surface due to the high propensity of nanoparticles towards agglomeration. These nanoparticles exhibit fluffy texture and have been observed to be evenly distributed on the surface of clay. These findings are in agreement with earlier studies [33, 42]. Similar, findings are observed in the case of TV-NC (Fig 8B), illustrating that structural integrity of VC remained undisturbed in the nanocomposite matrix. The TBC, HA, and MnFe_2_O_4_ nanoparticles possessing a spongy nature are clumped together in micro aggregates, with fairly even localization on the clay surface. These findings are in agreement with the earlier studies [40, 42].

The elemental composition of TK-NC and TV-NC is confirmed from EDX analysis (Fig 7). The EDX spectrum of TK-NC (Fig 8C) depicts oxygen as the chief element, accounting for 40.01%, corresponding to oxygen containing surface functional groups. Carbon is the second most abundant constituent element, accounting for 14.40% by weight, indicating the successful incorporation of TBC into the nanocomposite. The occurrence of Ca (11.73%) and P (7.11%) validated the formation of HA with a Ca/P molar ratio quantified as 1.65, which is in good agreement with earlier studies. The presence of Mn (4.93%) and Fe (10.04%) in a 1:2 ratio confirmed the formation of MnFe_2_O_4_, while the remaining elements of Al and Si in %weight of 5.43 and 5.27%, respectively, represent the presence of KC in the nanocomposites. The EDX spectrum of TV-NC is presented in Fig 8D. The O, C, Si, Al, and Mg with corresponding %weight of 39.97, 14.42, 3.94, 2.05, and 3.89%, respectively, are associated with TBC and VC presence. The fabrication of HA and MnFe_2_O_4_ is also confirmed by the occurrence of Ca/P and Mn: Fe in 1.64 and 1:2 ratios, respectively. A small quantity of S (1.39%) was also observed in the spectrum, appearing due to MnSO_4_ used in the nanocomposite synthesis. Hence, the EDX analysis in both cases confirmed the doping of TBC, HA, and MnFe_2_O_4_ on to clay structures.

### 3.2. Methylene blue test

**Fig 9:**
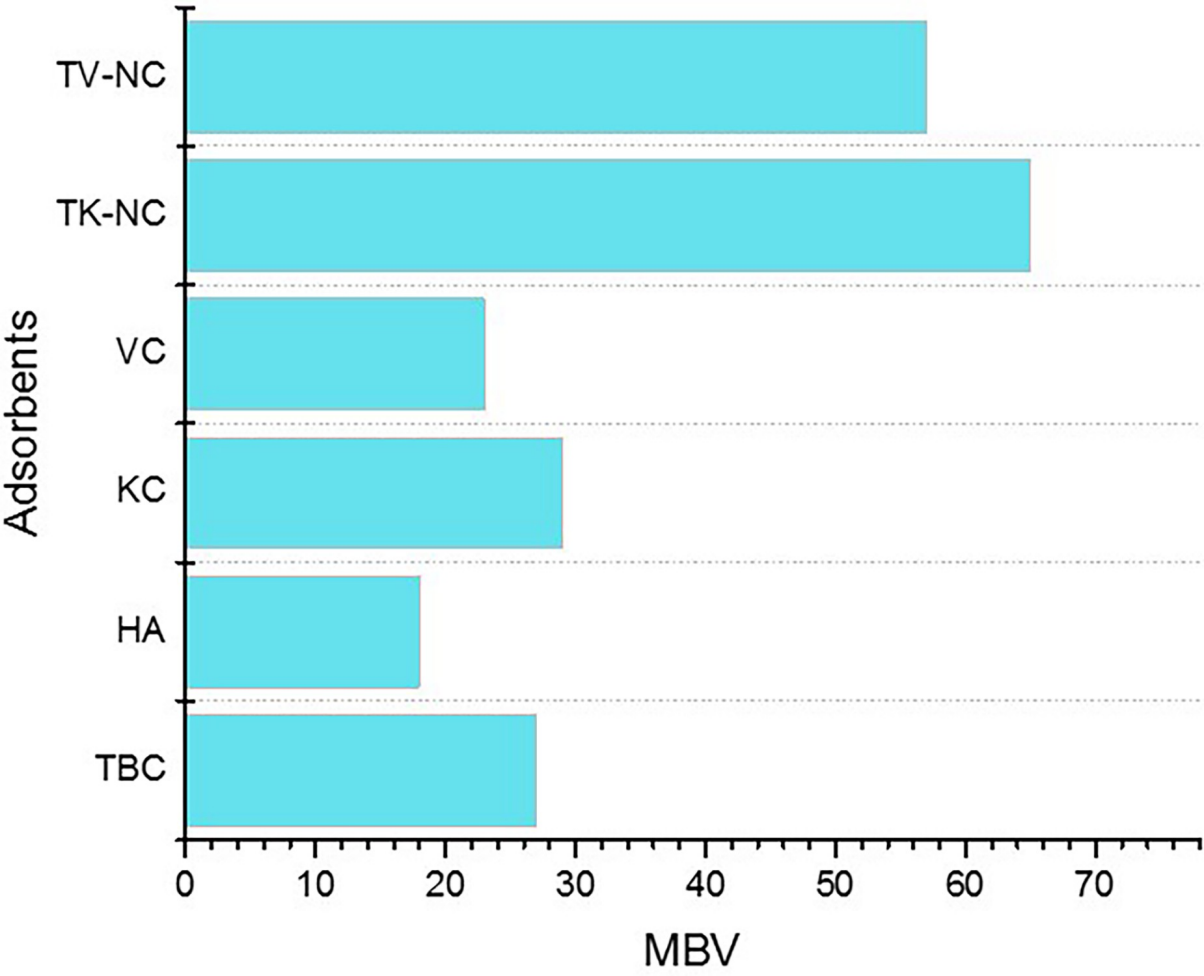
MBV of TK-NC and TV-NC, and their pristine components.

Methylene blue adsorption test is extensively employed as an indicator for evaluating the adsorption efficiency of any adsorbent, particularly carbon-based matrix, as this dye is often taken into account as a model contaminant due to difficulty of its degradation. It measures the adsorption performance of test adsorbent in terms of methylene blue value, which is the volume of standard methylene blue solution discolored with 0.1 g of adsorbent. The TK-NC, TV-NC, and their pristine components were subjected to methylene blue test to explore their adsorptive potential. The methylene blue values for all the test adsorbents are displayed in Fig 9. The methylene blue adsorption test was used as a screening test to select adsorbents with superior adsorption capacity for target heavy metals’ sorption studies. Both TK-NC and TV-NC exhibited 2–3 times superior scavenging activity for methylene blue than their pristine counterparts, and hence, based on these results, further studies were performed using these nanocomposites.

### 3.3. Adsorption of metal ions by nanocomposites (TK-NC and TV-NC)

TK-NC and TV-NC were examined for their efficacy against the removal of target heavy metals (Cu(II), Cr(VI) and Ni(II)) from aqueous solutions in batch mode as a function of varying experimental conditions (contact time, temperature, solution initial pH, adsorbent dose, and initial metal ion concentration).

#### 3.3.1. Effect of contact time and adsorption kinetics

**Fig 10:**
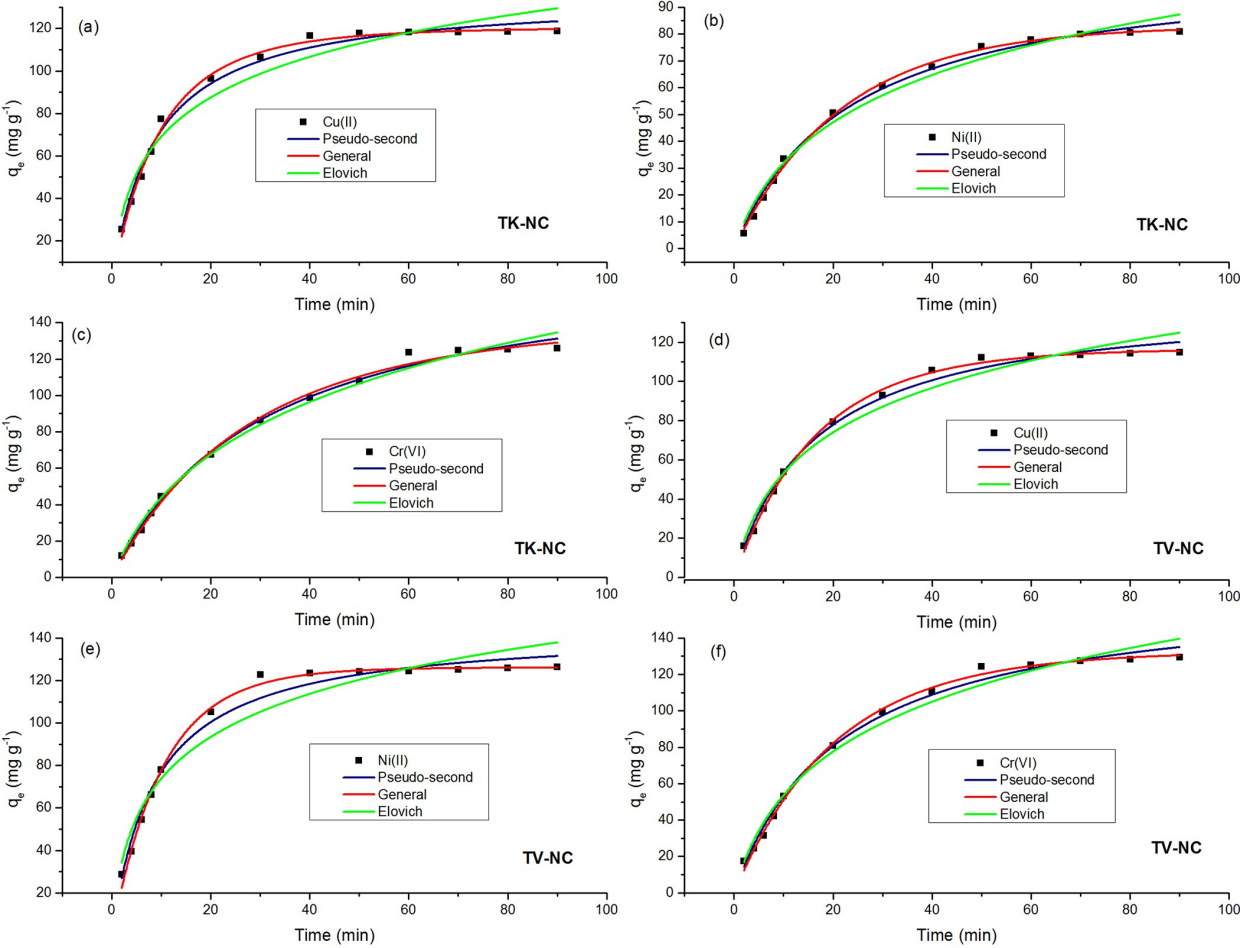
Effect of variation of time and fitting of kinetic models for Cu(II), Ni(II) and Cr(VI) adsorption on to TK-NC (a-c) and TV-NC (d-f) [T (25°C); pH (4.0); adsorbent dose (0.1 g); C_i_ (500 mg L^-1^)].

Contact time is an imperative function to be studied during the adsorption experiments [43]. The influence of contact time on adsorption of metal ions (Cu(II), Cr(VI), and Ni(II)) by TK-NC and TV-NC (Fig 10) was investigated by varying contact time (2–90 minutes). Rapid uptake of metals by both nanocomposites was observed during the initial phase due to availability of unbound active sites on the surface of nanocomposite for metal ions binding, resulting in fast adsorption process.

The metal ions adsorption on TK-NC and TV-NC increased with an increase in contact time, which was followed by a gradual decline in removal efficiency until equilibrium was achieved, and afterwards an insignificant rise in sorption capacities for both nanocomposites was noticed. The adsorption of metal ions onto TK-NC achieved equilibrium at 40 minutes for Cu(II), 50 minutes for Ni(II), and 60 minutes for Cr(VI) (Fig 10A–10C). Though, in the case of TV-NC, the equilibrium time of 50 minutes for Cu(II) and Cr(VI), and 30 minutes for Ni(II) was observed (Fig 10D–10F). Previous studies have registered a much longer time to attain optimal adsorption capacity, from 90 minutes to as long as 72h (S1 Table).

Adsorption kinetics describes the adsorption rate of solute onto the adsorbent and is very prominent function for evaluating the removal efficiency of an adsorbent. Different kinetics models (Pseudo-first-order, Pseudo-second-order, Elovich [44], and general-order [45] in non-linear form mentioned in Eqs (6–9), respectively, were fitted to experimental data (Fig 10).

$$q_{t}=q_{e}[1-exp(-k_{1}∙t)]$$ (6)

$$q_{t}=\frac{t{∙k}_{2}∙q_{e}^{2}\;}{1+{t∙k}_{2}∙q_{e}\;}$$ (7)

$$q_{t=}\;\frac{1}{b\;}[ln(a∙b∙t+1)]$$ (8)

$$q_{t=q_{e}-\frac{q_{e}}{\;{\left[k_{3\;}∙({q_{e\;}}^{\left(n-1\right)})∙t∙(n-1)+1\right]}^{\left(1/1-n\right)}}}$$ (9)

where *q*_*t*_ (mg g^-1^) is the concentration of adsorbent at time *t* (min), *q*_*e*_ (mg g^-1^) is the concentration at equilibrium, *k*_*1*_ (min^-1^) and *k*_*2*_ (g mg^-1^ min^-1^) and *k*_*3*_ (min^−1^ (g mg^−1^) ^n−1^) are the rate constants for pseudo-first, pseudo-second, and general orders, respectively, *a* (mg g^-1^ min^-1^) is the initial rate of adsorption, *b* (g mg^-1^) is the desorption constant, and n is the kinetic sorption order (n could be an integer or a fractional value).

The best fitting of model, as assumed from highest *R*^*2*^, lowest reduced chi-square and standard deviation values, and smallest difference between experimental (*q*_*e*,*exp*_) and calculated (*q*_*e*,*cal*_) adsorption capacities, comes out to be general order kinetics model for all six cases. The fitting parameters are presented in Table 1. The adsorption data did not converge to pseudo-first-order kinetic model; hence, the fitting and data are not presented in Fig 10 and Table 1. The suitability of adsorption data to general order kinetic model implicates that there is a shift in orders during the course of adsorption process [46]. In the case of Elovich model, the higher values of ‘a’ denote that the initial adsorption rate is high, while the lower values of ‘b’ indicate lower desorption rate [47]. In addition, higher value of ‘a’ and lower value of ‘b’ also implicate that adsorbent possesses bulk density of reactive sorption sites and high electron donor potential [48].

**Table 1:** Adsorption kinetics model fitting parameters.

Model	Parameter	TK-NC			TV-NC		
		Cr(VI)	Ni(II)	Cu(II)	Cr(VI)	Ni(II)	Cu(II)
Pseudo-seond-order	*k*_*2*_ (min^-1^)	1.81 x10^-4^	3.99 x10^-4^	8.40 x10^-4^	2.79x10^-4^	7.932 x10^-4^	4.280 x10^-4^
	*q*_*e cal*_ (mg g^-1^)	176.750	106.420	135.330	167.079	144.230	141.960
	*R*_*adj*_^*2*^	0.995	0.993	0.988	0.993	0.983	0.992
	*Reduced chi-square*	9.931	5.541	14.272	14.482	23.763	12.277
General order	*k*_*3*_ (min^-1^)	0.013	0.042	0.034	0.042	0.082	0.047
	*n*	1.213	1.022	1.234	1.034	1.036	1.051
	*q*_*e cal*_ (mg g^-1^)	140.770	83.090	120.460	132.590	126.069	116.520
	*R*_*adj*_^*2*^	0.996	0.997	0.994	0.997	0.996	0.998
	*Reduced chi-square*	8.856	2.247	6.660	6.650	5.992	3.290
Elovich	*a* (mg g^-1^ min^-1^)	6.803	5.645	29.097	10.138	31.505	12.061
	*b* (g mg^-1^)	0.019	0.033	0.035	0.022	0.033	0.027
	*R*_*adj*_^*2*^	0.990	0.981	0.949	0.980	0.938	0.972
	*Reduced chi-square*	19.577	15.469	60.079	38.92	48.118	40.735
Intra-particle diffusion	*k*_*id1*_ (mg/g.min^0.5^)	16.261	13.912	26.514	16.909	26.990	17.587
	*C*_*i1*_	10.226	0.203	2.356	1.776	5.220	4.289
	*R*_*1adj*_^*2*^	0.954	0.981	0.993	0.936	0.971	0.977
	*k*_*id2*_ (mg/g.min^0.5^)	16.342	10.886	12.599	18.444	21.159	14.243
	*C*_*i2*_	15.938	0.749	38.350	3.596	10.989	15.793
	*R*_*2adj*_^*2*^	0.995	0.985	0.981	0.986	0.1	0.992
	*k*_*id3*_ (mg/g.min^0.5^)	1.267	2.373	0.600	2.214	0.895	1.085
	*C*_*i3*_	113.986	59.198	113.262	108.596	117.792	104.524
	*R*_*3adj*_^*2*^	0.976	0.885	0.825	0.965	0.989	0.995

The adsorption kinetics were further investigated to identify the rate-controlling steps occurring during the adsorption process by using the intra-particle diffusion model [43], which is represented by Eq (10).

$$q_{t}=k_{id}t^{0.5}+C_{i}$$ (10)

where t (min) is the time, the slope kid (mg/g.min^0.5^) is the intra-particle diffusion rate constant, q_t_ (mg g^-1^) is the adsorption capacity at time (t), and C_i_ represents the thickness of boundary layer of stage *i*. A higher value of C_i_ indicates a greater boundary layer effect. If the intra-particle diffusion is the sole limiting factor, the plotted lines will pass through the origin (C_i_ = 0). However, if there is some involvement of external mass transfer, the lines will deviate from origin. The obtained fitting parameters and plots for intra-particle diffusion model are presented in Table 1 and S2 Fig. The investigations employing intra-particle diffusion model implies, in all six cases, different sorption phenomena were occurring as the plot fitted to three straight lines and the plotted lines did not pass through the origin (C_i_ ≠ 0), suggesting a multi-step adsorption process [46].

#### 3.3.2. Effect of temperature

**Fig 11:**
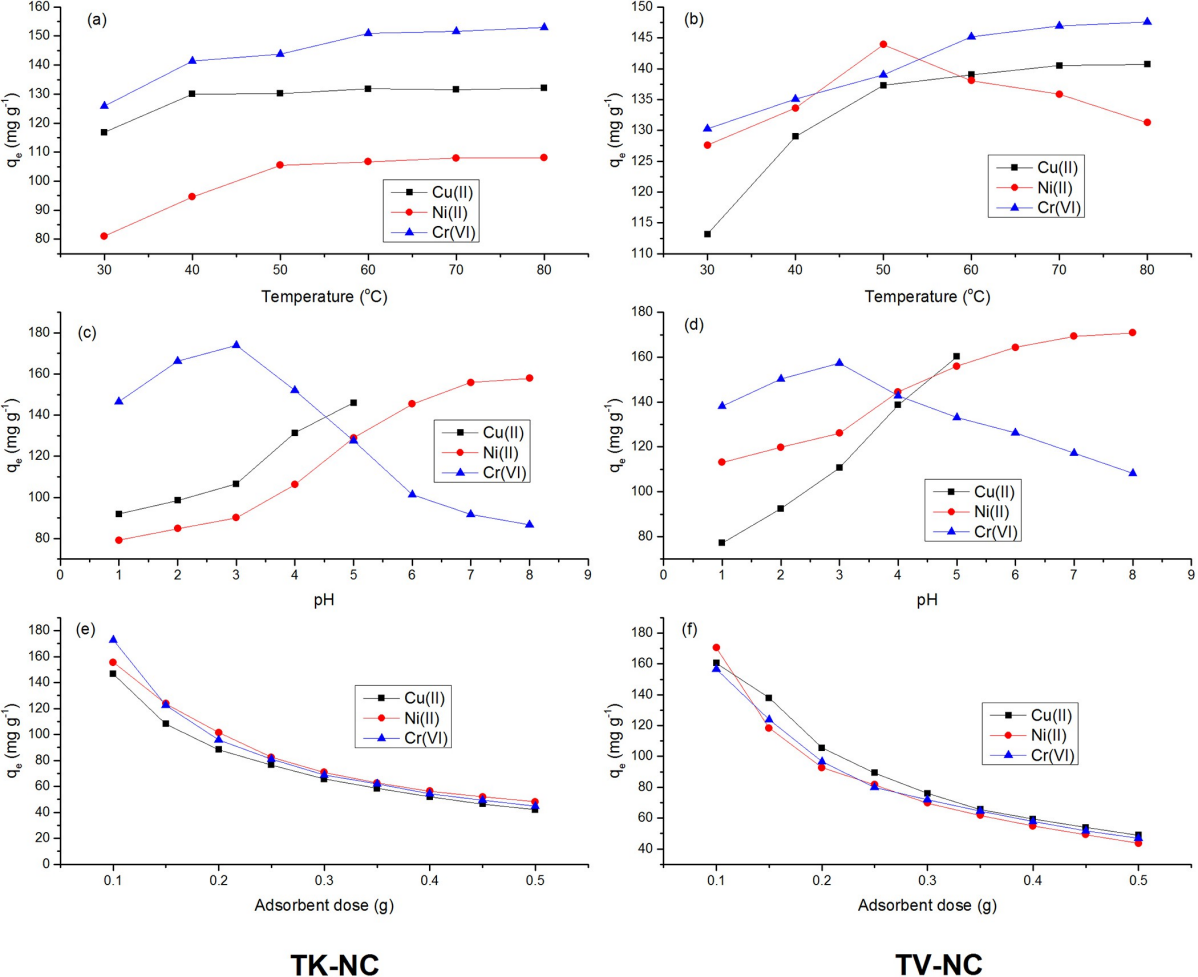
Effect of variation of temperature (a, b), initial pH (c, d) and adsorbent dose (e, f) for adsorption Cu(II), Ni(II) and Cr(VI) on to TK-NC and TV-NC, respectively. [TK-NC: t (40, 50, 60 min); pH (4.0); adsorbent dose (0.1 g); C_i_ (500 mg L^-1^), TV-NC: t (50, 30, 50 min); pH (4.0); adsorbent dose (0.1 g); C_i_ (500 mg L^-1^) for Cu(II), Ni(II), and Cr(VI), respectively] [TK-NC: t (40, 50, 60 min); T (50, 50, 60°C); adsorbent dose (0.1 g); C_i_ (500 mg L^-1^), TV-NC: t (50, 30, 50 min); T (60, 50, 60°C); adsorbent dose (0.1 g); C_i_ (500 mg L^-1^) for Cu(II), Ni(II), and Cr(VI), respectively] [TK-NC: t (40, 50, 60 min); T (50, 50, 60°C); pH (5.0, 7.0, 3.0); C_i_ (500 mg L^-1^), TV-NC: t (50, 30, 50 min); T (60, 50, 60°C); pH (5.0, 7.0, 3.0); C_i_ (500 mg L^-1^) for Cu(II), Ni(II), and Cr(VI), respectively].

To investigate the influence of temperature, a set of batch experiments were performed by changing temperature from 30 to 80°C (Fig 11A, 11B). The experimental data showed that metal sorption by nanocomposites was found to be highly temperature dependent and continuously augmented with increments in reaction temperature owing to thermal activation of metal ions, which results in fast mobility of metal ions, therefore leading to high sorption performance [49]. The adsorption of Cu(II) and Ni(II) onto TK-NC improved incrementally with an upsurge in temperature up to 50°C, and for Cr(VI), it increased up to 60°C; afterwards, further increments in temperature resulted in an insignificant increase in metal adsorption. Similarly, in the case of TV-NC, adsorption of Cu(II) and Cr(VI) enhanced up to 60°C, and for Ni(II), it augmented up to 50°C, which was also followed by a very negligible change in metal adsorption. The increment in metal removal capacity by both nanocomposites with increase in temperature revealed the endothermic nature of sorption process, which is in agreement with previously reported studies. The higher Cr(VI) uptake was registered at elevated temperatures on zinc-iron ferrate anchored *Macadamia* nutshell biochar and montmorillonite modified peanut shell biochar [50, 51]. Similarly, temperature increase has been found to encourage the rejection efficiency of Ni(II) onto Magnetic hydroxyapatite doped polydopamine and Ni(II) and Cu(II) onto magnetic *Zea mays* based biochar [49, 52]. In another study, the uptake of Cu(II) onto magnetic nanoparticles modified chitosan grafted graphene oxide also exhibited an endothermic nature of sorption process [53].

Thermodynamic parameters, i.e. standard free energy change (Δ*G°*), standard enthalpy change (Δ*H°*) and standard entropy change (Δ*S°*), are calculated using the following equations:

$$\Delta G=-RTlnK_{C}$$ (11)

$$Where,\;K_{C}=\frac{C_{S}}{C_{e}}$$ (12)

$$lnK_{C}=(\frac{{\Delta S}^{{}^{\circ}}}{R})-(\frac{{\Delta H}^{{}^{\circ}}}{RT})$$ (13)

where, *K*_*C*_, *C*_*s*_ and *C*_*e*_ are equilibrium constant, equilibrium concentration of the adsorbate sorbed on the adsorbent in mg L^-1^, and equilibrium concentration of adsorbate in the solution in mg L^-1^. *R* is ideal gas constant which value is 8.314 J mol^-1^ K^-1^ and *T* is the adsorption temperature in Kelvin (K).

The plot of ln*K*_*C*_ vs *T*^*-1*^ for the adsorption of Cu(II), Ni(II), and Cr(VI) on TK-NC and TV-NC (S3 Fig) is used to calculate Δ*H°* and Δ*S°* from slope and intercept, respectively (Table 2). The values show that the adsorption of all metal ions on to TK-NC and TV-NC is endothermic (+*ΔH°*) but points to disorderness around the nanocomposite surface owing to the strong affinity of metal ions with adsorbent as reflected by +*ΔS*° value [54]. The positive values of *ΔG°* for Cu(II), Ni(II), and Cr(VI) by TK-NC and TV-NC suggest that the adsorption of all metal ions on both nanocomposites is non-spontaneous. These findings are in accordance with the previous work in the support of non-spontaneity of adsorption of metal ions (Cu(II), Pb(II), Z(II), ad Cd(II)) on olive branches derived activated carbon at the temperature interval (303–323 K) [54].

**Table 2:** Thermodynamic parameters’ values for adsorption of Cu(II), Ni(II), and Cr(VI) on TK-NC and TV-NC.

Nano-adsorbents	*Metal ions*	*T* (K)	*K*_*C*_	Δ*G°* kJ mol^-1^	Δ*H°* kJ mol^-1^	Δ*S°* kJ mol^-1^K^-1^
**TK-NC**	Cu(II)	303	0.495	1.771	3.072	0.005
		313	0.549	1.560		
		323	0.550	1.604		
		333	0.558	1.615		
	Ni(II)	303	0.312	2.932	4.761	0.006
		313	0.340	2.810		
		323	0.365	2.710		
		333	0.368	2.770		
	Cr(VI)	303	0.608	1.253	5.850	0.020
		313	0.695	0.947		
		323	0.707	0.932		
		333	0.762	0.751		
**TV-NC**	Cu(II)	303	0.508	1.710	4.568	0.011
		313	0.574	1.443		
		323	0.581	1.460		
		333	0.615	1.354		
	Ni(II)	303	0.588	1.334	3.241	0.006
		313	0.618	1.251		
		323	0.679	1.041		
		333	0.647	1.210		
	Cr(VI)	303	0.606	1.260	3.665	0.008
		313	0.633	1.189		
		323	0.660	1.117		
		333	0.692	1.020		

#### 3.3.3. Effect of pH

The pH of solution is a significant experimental parameter that is essential to be studied in adsorption experiments as it controls the uptake of metal ions onto the adsorbent surface. It influences the adsorbents’ surface charge and speciation of adsorbent and adsorbate molecules in aqueous solution [55, 56]. In order to investigate the effect of pH variation on metal ions adsorption by TK-NC and TV-NC, batch experiments were carried out at various pH values ranging from 1.0 to 8.0 for Ni(II) and Cr(VI), and 1.0 to 5.0 for Cu(II). The sorption experiments at pH >5.0 (in the case of Cu(II) and pH > 8.0 (in the case of Ni(II) and Cr(VI)) were not conducted due to the formation of insoluble metal precipitates [13, 57]. For determining the impact of solution pH on the adsorption of metal ions by nanocomposites, 50 ml of each metal solution (500 mg L^-1^) was agitated for predetermined optimum time period and temperature with 0.1 g of adsorbent at altering pH levels. The data is presented in Fig 11C, 11D.

In acidic medium, hydrogen chromate (HCrO_4_^-^) ions are the most predominant form of Cr(VI), and when the pH moves towards basic medium, chromate ions (CrO_4_^2-^) are the most prevalent species to occur in the aqueous system [58]. The adsorption of Cr(VI) was enhanced with a reduction in pH (acidic), and the maximum Cr(VI) adsorption capacities of 173.94 and 157.35 mg g^-1^ were observed at pH 3.0 for TK-NC and TV-NC, respectively. If pH < pH_(PZC)_, the charge on the nanocomposite surface would be positive, and if pH > pH_(PZC)_, it would be negative [43]. In this study, at pH < pH_(PZC)_ (i.e., 4.65 for TK-NC and 4.15 for TV-NC) (S4A and S4B Fig), the surface of nanocomposites was dominated by the positive charge owing to the presence of excessive H^+^ ions in the surrounding aqueous media, encouraging the adsorption of Cr(VI) as HCrO_4_^-^. With an increase in pH (above pH_(PZC)_) the positive charge density gradually reduces due to an increase in the density of OH^-^ ions, and the nanocomposites’ surface get an anionic charge, resulting in electrostatic repulsion between negatively charged adsorbents’ surface and Cr(VI) anionic species, which leads to poor metal adsorption performance [59].

The uptake capacity of 23.85 mg g^-1^ for Cr(VI) using magnetic modified *Astragulus membranaceus* derived biochar was achieved at pH 2.0 [60]. In another study, the acidic pH (3.0) favored the adsorption of Cr(VI) on Fe_3_O_4_ doped hydroxyapatite encapsulated alginate beads, with maximum removal capacity recorded as 29.32 mg g^-1^ [42]. In the pH range of 3.0–4.0, Cu^2+^ and CuOH^+^ ions are the most prevalent species of Cu(II), whereas at pH (>5.0), Cu(II) exists as insoluble precipitates of Cu(OH)_2_ [57]. The uptake of Cu(II) enhanced with increase in pH, and the maximum adsorption of Cu(II) (165.71 and 172.73 mg g^-1^) onto TK-NC and TV-NC, respectively, were recorded at pH 5.0. At pH ≥ 5.0, Ni^2+^ is the most prevalent form of Ni(II) in aqueous media [61], and very high alkaline pH is not recommended due to formation of Ni(OH)_2_ precipitates. At pH 7.0, the highest uptake of Ni(II) onto TK-NC and TV-NC occurred, with removal capacities of 176.81 and 166.62 mg g^-1^, respectively. At low pH<pH_(PZC)_, the nanocomposite surface becomes positively charged due to presence of excessive H^+^ ions in the aqueous solution, which impede the binding of Cu(II) and Ni(II) on its surface owing to the existence of electrostatic repulsion between H^+^ and metal ions. With the increment in pH above pH_(PZC)_, the character of oxygen bearing surface functionalities (COOH, OH) present on the surface of nanocomposite alters, and the surface gets an anionic charge, which results in electrostatic attraction between negatively charged adsorbents’ surface and Cu(II) and Ni(II) ionic species, leading to high metal uptake activity [55, 56]. The similar response was evidenced in the present study, in which low uptake of Cu(II) and Ni(II) ions onto the fabricated nanocomposites was observed in acidic medium and began to increase with increment in pH, and the highest removal efficiencies were noticed in pH range of 5.0–7.0. Hydrous zirconium oxide nanoparticles modified vermiculite was employed for abatement of Cu(II) from aqueous solution, and a considerable high sorption efficiency of 85% for Cu(II) was recorded at a pH of 5.0 [62]. Ansari *et al*. reported that the optimized pH for removal of Cu(II) (~70%) using Fe_3_O_4_ incorporated hydroxyapatite modified β-cyclodextrin was 6.0 [55]. The removal efficiency of >90% for Ni(II) using hydroxyapatite functionalized magnetic polydopamine was obtained at pH 6.0 [52]. In another study, the highest adsorption of Ni(II) (8.20 mg g^-1^) on magnesium/zinc ferrite nanoparticles was recorded at pH 7.0 [56].

#### 3.3.4. Effect of adsorbent dose

The influence of adsorbent dose on metal ions scavenging was explored in batch studies by altering its dose (0.1–0.5 g) at optimum time, temperature, and pH and using 500 mg L^-1^ of each metal ion solution. By varying adsorbent dosage from 0.1 to 0.5 g, a noticeable increase in the % RE from (66.37–87.60%), (72.38–92.08%), and (70.78–94.06%) for Cu(II), Cr(VI), and Ni(II), respectively, by TK-NC was observed. In the case of TV-NC, % RE for Cu(II), Cr(VI), and Ni(II) incremented from (69.12–85.37%), (76.74–88.73%), and (66.45–91.82%), respectively. The augmented % RE of metal ions is attributed to increase in the number of active adsorption sites and surface functional groups for uptake of metal ions with the increase in adsorbent dose. At 0.1 g of adsorbent dose, TK-NC exhibited highest adsorption capacity of 146.64, 172.64 and 155.50 mg g^-1^ for Cu(II), Cr(VI) and Ni(II), respectively, whereas TV-NC has been found to remove 160.53mg g^-1^ (Cu(II)), 156.67 mg g^-1^ (Cr(VI)) and 170.47 mg g^-1^ (Ni(II)) at the same adsorbent dose (Fig 11E, 11F), following a gradual decrease in adsorption capacity of both nanocomposites with further increase in dose above 0.1 g. The maximum adsorption for Cu(II), Ni(II), and Cr(VI) was recorded at 0.1 g adsorbent dose of TK-NC and TV-NC, so it was considered as optimum dose for adsorption of metal ions from aqueous solution by both nanocomposites. However, for further increment in adsorbent dosage beyond the 0.1 g, the substantial reduction in uptake capacity by both nanocomposites for all metal ions was noticed. This is ascribed to the fact that an increase in adsorbent dose augments the density of vacant reactive sites, which initially results in rapid scavenging activity, but the sorption activity is compromised at adsorbent dose > 0.1 g since a bulk number of active sorption sites are left vacant because the adsorbent dose is incremented at fixed concentration of metal ions [63].

For instance, Ni(II) uptake capacity using FeOOH/Fe_3_O_4_ nanoparticles decorated sewage sludge biochars decreased with an increase in adsorbent dose (0.01–0.1 g L^-1^), and 0.01 g was evaluated as optimum dosage with a maximum uptake capacity of 35.50 mg g^-1^ [64]. Ge *et al*. identified the effect of variation in adsorbent dosage (0.3–1.8 g L^-1^) on Cu(II) removal by using porous geopolymeric nanospheres. The highest Cu(II) removal of 38.55 mg g^-1^ was achieved at an adsorbent dose of 0.035g/ 50 ml and reduced with further increase in adsorbent dosage [65]. Zhu *et al*. employed Mn-Fe oxide impregnated corn straw biochar for Cr(VI) adsorption and estimated that the Cr(VI) uptake efficiency improved from 71.13–91.79% with an increase in adsorbent dose from 1.0–2.0g L^-1^. However, further increase in dosage up to 6.0 g L^-1^ registered an insignificant improvement in removal efficiency) [63].

#### 3.3.5. Effect of initial metal ion concentration and adsorption isotherm

The effect of initial metal ions concentration was investigated by altering concentrations of Cu(II), Cr(VI), and Ni(II) in the range of 300–1000 mg L^-1^ while maintaining the rest of the adsorption parameters constant as optimized in the preceding experiments.

**Fig 12:**
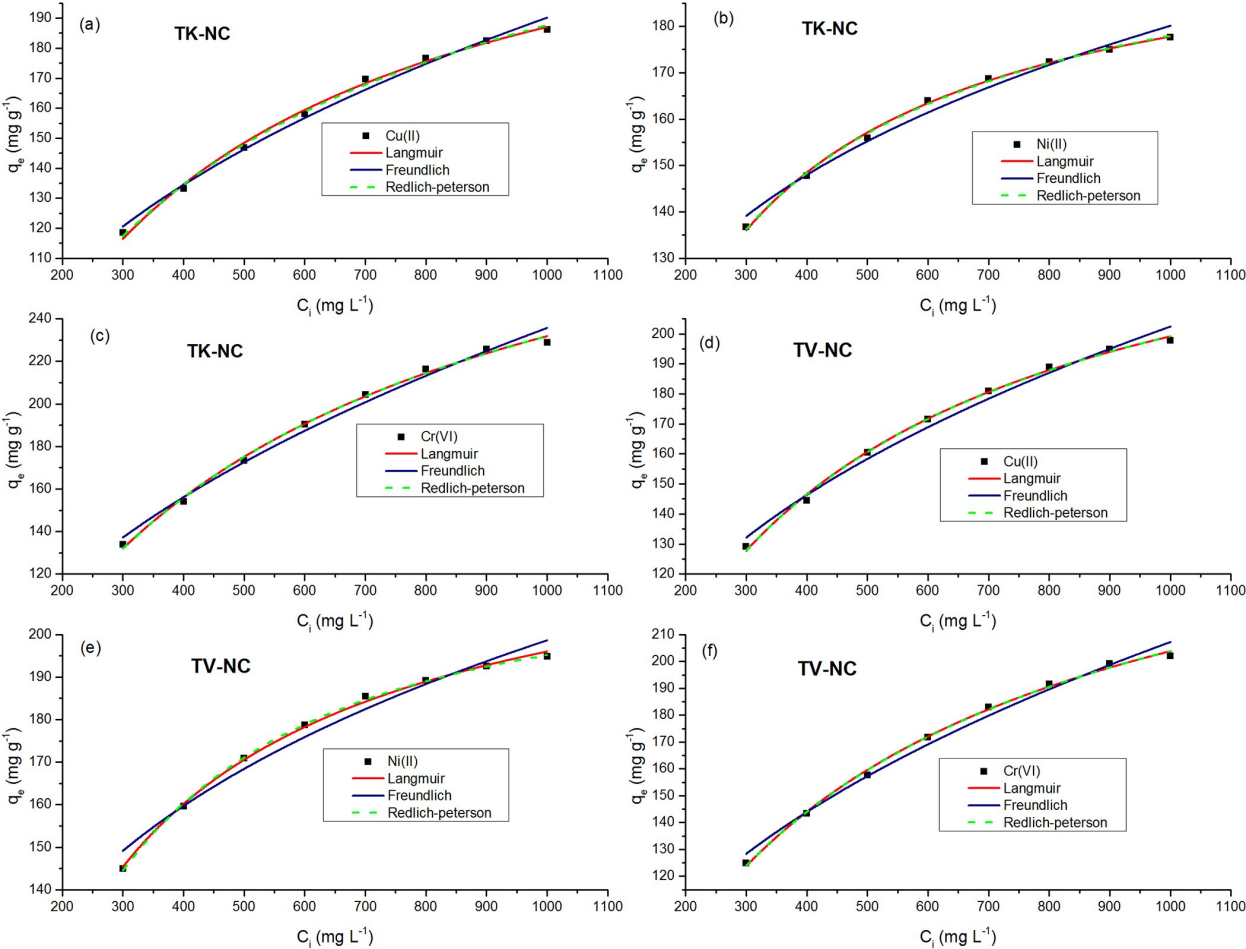
Effect of variation of initial metal ion concentration and fitting of isotherm models for Cu(II), Ni(II), and Cr(VI) adsorption on to TK-NC (a-c) and TV-NC (d-f) [TK-NC: t (40, 50, 60 min); T (50, 50, 60°C); pH (5.0, 7.0, 3.0); adsorbent dose (0.1 g), TV-NC: t (50, 30, 50 min); T (60, 50, 60°C); pH (5.0, 7.0, 3.0); adsorbent dose (0.1 g), for Cu(II), Ni(II), and Cr(VI), respectively].

The solute transfer driving force enhances as the initial metal ions concentration increments. A higher initial concentration of metal ions delivers a very strong driving impetus of the concentration gradient, which improves metal ions binding on the surface of adsorbent, resulting in a higher uptake capacity. The increase in metal ion concentration from 300 to 1000 mg L^-1^ resulted in enhancement of adsorption capacity of both nanocomposites (Fig 12).

The uptake of Cr(VI) by magnesium ferrite modified activated charcoal increased with an increase in initial metal ion concertation (100–500 mg L^-1^), and maximum Cr(VI) sorption capacity was enhanced up to 500.0 mg g^-1^ [66]. In another study, the Cu(II) scavenging capacity of hydroxyapatite doped *Undaria pinnatifida* root biochar was augmented with an increment in initial metal concentration (10–1000 mg L^-1^), with highest Cu(II) sorption capacity being registered as 99.01 mg g^-1^ [67]. Zhu *et al*. performed experiments to investigate the effect of initial metal concentration on the removal of Cr(VI) by Mn-Fe oxide impregnated corn straw biochar. With an increment in Cr(VI) concentrations from 25–800 mg L^-1^, the metal removal capacity of nanocomposite was augmented from 12.43 to 118.03 mg g^-1^ [63].

To get deep insight into the nature of sorption systems, it is required to conduct the study of equilibrium isotherms. Different isotherm models like Langmuir, Freundlich, and Redlich-Peterson in non-linear form were applied for the analysis of experimental data.

Langmuir model [45] proposes that adsorption takes place on homogenous sites via monolayer adsorption with no interaction between adsorbate-adsorbent molecules.


$$q_{e}=\frac{q_{m}∙K_{l}∙C_{e}}{1+K_{l}∙C_{e}}$$ (14)

The *q*_*m*_ is the maximum adsorption capacity of the adsorbent (mg g^-1^), *C*_*e*_ is the concentration of adsorbate at equilibrium (mg L^-1^), *q*_*e*_ represents the sorption capacity at equilibrium (mg g^-1^), and *K*_*l*_ is Langmuir constant (L mg^-1^) associated with the energy of sorption, indicating how favorable thermodynamically the sorption of the adsorbate molecule is on the surface of adsorbent. The *K*_*l*_ can be employed for calculating the Langmuir partitioning factor *(*dimensionless constant), *R*_*L*_, using the following equation:

$$R_{L}=\frac{1}{\left(1+K_{l}∙C_{0}\right)}$$ (15)

Here, C_0_ represents the initial concentration of adsorbate (mg L^-1^). The value of *R*_*L*_ predicts about the nature of sorption process to be like; if *R*_*L*_ = 0 (irreversible); 1> *R*_*L*_> 0 (favorable); and 1< *R*_*L*_ (unfavorable).

The Freundlich isotherm [44] addresses the heterogeneity of adsorbent surface and obeys multilayer adsorption of adsorbate molecules on adsorbent surface.

$$q_{e}=K_{F}\;\left(C_{e}^{\frac{1}{n_{F}}}\right)$$ (16)

where *K*_*F*_ ((mg g^-1^) (L mg^-1^)^1/n^) represents the intensity of sorption and *n*_*F*_ is the Freundlich dimensionless constants, which measures the deviation of sorption process from linearity. If the value of 1/*n*_*F*_ is approaching 0, then it is indicative of the heterogeneous nature of the system, and if it is very close to 1, it specifies homogeneity of the system. The value of 1/*n*_*F*_ ranging between 0 to 1 also implies the favorability of adsorption process.

The Redlich-Peterson (R-P) isotherm [47] is a 3 parameters model that delineates the homogenous and heterogeneous behaviors of sorption process by incorporating the features of Langmuir and Freundlich models.


$$q_{e=}\frac{K_{R}∙C_{e}}{\left(1+a∙{C_{e}}^{g}\right)}$$ (17)

*K*_*R*_ is the R-P constant (L g^-1^), *a* is R-P isotherm constant (L mg^-1^), and *g* is an exponent that has a value in the range of 0 to 1. If the value of *g* is approaching 0, then it is reduced to Freundlich isotherm equation, and if its value is approaching 1, then it is inclined to Langmuir isotherm. *Ce* is the concentration of adsorbate at equilibrium (mg L^-1^), and *q*_*e*_ is the adsorption capacity at equilibrium (mg g^-1^).

Table 3 lists the isotherm model parameters, which shows that the Langmuir and R-P isotherm models exhibited well-fitting to experimental data (Fig 12) as compared to Freundlich isotherm model.

**Table 3:** Adsorption isotherm model fitting parameters.

Model	Parameter	TK-NC			TV-NC		
		Cr(VI)	Ni(II)	Cu(II)	Cr(VI)	Ni(II)	Cu(II)
Langmuir	*K*_*l*_ (L mg^-1^)	0.002	0.007	0.003	0.003	0.006	0.003
	*q*_*m*_ (mg g^-1^)	343.050	204.680	252.650	281.820	230.340	261.870
	*R*_*L*_	0.487	0.233	0.408	0.435	0.260	0.385
	*R*_*adj*_^*2*^	0.996	0.998	0.995	0.997	0.998	0.997
	*Reduced chi-square*	4.625	0.489	2.579	2.068	0.634	1.585
Freundlich	*K*_*F*_ (mg g^-1^ (mg L^-1^)^-1/nF^)	10.548	40.938	13.933	13.274	38.331	17.497
	*n*_F_	2.223	4.663	2.643	2.514	4.198	2.821
	*R*_*adj*_^*2*^	0.987	0.980	0.989	0.987	0.969	0.985
	*Reduced chi-square*	15.725	4.033	6.631	10.239	9.582	9.232
R-P	*K*_*R*_ (L g^-1^)	0.709	1.449	0.847	0.749	1.106	0.845
	*g*	1.00	0.982	0.916	0.988	1.00	0.991
	*a* (L mg^-1^)	0.002	0.008	0.006	0.003	0.003	0.004
	*R*_*adj*_^*2*^	0.995	0.998	0.995	0.997	0.999	0.997
	*Reduced chi-square*	5.548	0.450	2.489	2.473	0.275	1.897

However, for adsorption of all metal ions on both nanocomposites, the R-P isotherm model holds best among others, as justified by highest R^2^ and reduced chi-square. The K_l_ values in Langmuir isotherm model ranged between 0–1, which indicates a good interaction between the adsorbent and metal ions. In the case of both nanocomposites, the values of R_L_ ranged between 0.233–0.487, which are less than unity, which affirms the favorability of sorption of metal ions on the nanocomposites surface. The favorability of adsorption of all metal ions on TK-NC and TV-NC was also confirmed by n_*F*_ values (2.223–4.663) obtained from Freundlich isotherm model, which are in the range of 1–10. The 1/ n_*F*_ values ranging between (0.215–0.450) showed the heterogeneous nature of TK-NC and TV-NC surfaces. For all metal ions investigated, the R-P exponent *g* values (0.916–1.00) have approached unity, indicating that R-P has reduced to Langmuir isotherm. This inferred that the adsorption of all metal ions took place via monolayer coverage on heterogeneous surfaces of both nanocomposites with a relatively uniform distribution of binding sites [68]. These findings are in good agreement with previous findings where adsorption of metal ions on various hybrid nanocomposite matrices obeyed Langmuir isotherm [52, 55]. The adsorption capacities for TK-NC and TV-NC as obtained from Langmuir adsorption model follow the following order: Cr (343.05 mg g^-1^) > Cu (252.65 mg g^-1^) > Ni (204.68 mg g^-1^) and Cr (281.82 mg g^-1^) > Cu (261.87 mg g^-1^) > Ni (230.34 mg g^-1^), respectively. For Ni(II) and Cu(II), the removal capacity of TV-NC is better than that of TK-NC, while for Cr(VI), V-NC performed better. The current study demonstrated that the prepared nanocomposites (TK-NC and TV-NC) showed enhanced adsorption capacity for all three heavy metals (Cu(II), Cr(VI), and Ni(II)), which is much greater than already reported adsorbents (S1 Table), with an added advantage of consuming less time to remove these contaminants and requiring less stringent conditions for the adsorption, making the adsorbent process cost-effective.

### 3.4. Mechanism of adsorption of metals by nanocomposites

The FTIR spectra of TK-NC and TV-NC after metal adsorption were examined to explore the plausible removal mechanism of metal ions by both nanocomposites. The shifting in band positions of characteristic surface functional groups (-OH, C = C, C = O, PO_4_^3-^, Si-O, Al-OH, Si-O-Al, and Mg/Al-OH) for TK-NC and TV-NC after metal adsorption was noticed (S2 Table), which suggest the involvement of these corresponding surface functional groups in the adsorption of Cu(II), Ni(II), and Cr(VI) on the surface of both nanocomposites.

Both nanocomposites consist of different phases as identified by XRD (Fig 7C, 7D), which provide multiple adsorption sites within the nanocomposite for uptake of metal ions. These active sites lead to multiple adsorption phenomena and reactions, which can be explained as follows:

The metal ions (denoted as M^+^) might be chelated with–OH and–COOH functionalities from biochar present on the surface of both nanocomposites, as represented in Eqs (18–19), as suggested by [69].


$$‐COOH+M^{+}\to ‐COO^{‐}\ldots ..M^{+}+H^{+}$$ (18)

$$R‐OH+M^{+}\to R‐O^{‐}\ldots ..M^{+}+H^{+}$$ (19)

The removal of metals from aqueous media occurred via cation exchange between metal ions and Ca(II) of HA along with dissolution of HA, followed by chemical precipitation of metal ions as corresponding metal phosphate (M_10_(PO_4_)_6_(OH)_2_), and the release of proton in aqueous media from ≡PO-H functional site of HA and its subsequent compensation by metal ion to form ≡PO-M^+^ is given in Eqs (20–22) as suggested by [70].

Cation exchange;

$$Ca_{10}{\left(PO_{4}\right)}_{6}{\left(OH\right)}_{2}+M^{2+}\to Ca_{10‐a}M_{a}{\left(PO_{4}\right)}_{6}+aCa^{2+}+H_{2}O$$ (20)

Dissolution of HA;

$$Ca_{10}{\left(PO_{4}\right)}_{6}{\left(OH\right)}_{2}+14H^{+}\to 10Ca^{2+}+6HPO_{4}{}^{‐}+2H_{2}O$$ (21)

M_10_(PO_4_)_6_(OH)_2_ formation;

$$2H_{2}O+10M^{2+}+6HPO_{4}{}^{‐}\to 4H^{+}+M_{10}{\left(PO_{4}\right)}_{6}{\left(OH\right)}_{2}$$ (22)

The removal mechanism of metal ions by MnFe_2_O_4_ nanoparticles as proposed by Tatarchuk *et al*. [56] suggested that the surface of MnFe_2_O_4_ nanoparticles gets protonated under acidic pH and attained an anionic charge in alkaline conditions, which encouraged the binding of metal ions on its surface via electrostatic attraction (Eqs 23 and 24).


$$=X–OH+H^{+}\to =X^{+}\;(under\;acidic\;conditions)$$ (23)

$$=X–OH+OH^{‐}\to =X–O^{‐}\;(under\;basic\;conditions)\;[X=MnFe_{2}O_{4}]$$ (24)

Moreover, the silanol and aluminol groups of clays might interact with metal ions to form corresponding metal hydroxides (Eq 25) [71].


$$2Y–O^{‐}+M^{2+}\to {\left(YO\right)}_{2}M\;[Y=Si\;or\;Al]$$ (25)

Rapid uptake during the initial several minutes was attributed to electrostatic interaction and ion exchange, and later on the adsorption was governed by surface complexation and chemical precipitation.

## 4. Conclusion

The kaolinite and vermiculite-based nanocomposites (i.e., manganese ferrite doped hydroxyapatite/kaolinite/biochar (TK-NC) and manganese ferrite doped hydroxyapatite/vermiculite/biochar (TV-NC)) with crystallite sizes ranging from 2.55–5.94 nm were synthesized using a facile one pot synthesis approach for abatement of multiple heavy metals from water. The results of powder XRD and EDX analysis confirmed the successful integration of clay, hydroxyapatite, tea waste biochar, and manganese ferrite into the nanocomposite matrix. The SEM analysis exhibited that all the components of nanocomposites retained their structural integrity and were evenly localized on the surface of nanocomposite. TK-NC and TV-NC exhibited efficient removal of selected metal ions within the contact time of 30–60 min at 0.1 g adsorbent dose, temperature (50–60°C), and pH (3.0 (Cr(VI)), 5.0 (Cu(II)), and 7.0 (Ni(II))). The adsorption of metal ions, as obtained from the Langmuir isotherm, followed the following order: Cr (343.05 mg g^-1^) > Cu (252.65 mg g^-1^) > Ni (204.68 mg g^-1^) for TK-NC, and Cr (281.82 mg g^-1^) > Cu (261.87 mg g^-1^) > Ni (230.34 mg g^-1^) for TV-NC. TV-NC exhibited better adsorption activity for Cu(II) and Ni(II) as compared to TK-NC, while for Cr(VI), TK-NC surpassed TV-NC. The adsorption of metal ions by TK-NC and TV-NC was predominantly accredited to ion exchange, electrostatic interaction, surface complexation, and chemical precipitation. The employment of TK-NC and TV-NC in wastewater purification signifies an economically viable and environment-friendly alternative since it provides an innovate and green perspective of reutilizing tea waste and natural clays of low economic value to fabricate plausibly efficient nanocomposites, which have registered profound Cu(II), Cr(VI), and Ni(II) removal from aqueous systems in addition to being less time consuming and requiring less stringent conditions for adsorption. Thus, TK-NC and TV-NC can be used as potential nanocomposites for abatement of heavy metals from aqueous environments.

## Supporting information

S1 Fig FT-IR of nanocomposites TK-NC (a-c) and TV-NC (d-f) after adsorption of metal ions.
(TIF)

S2 Fig Fitting of intra-particle diffusion model (q_t_ vs t^0.5^) to adsorption of Cu(II), Ni(II), and Cr(VI) on to TK-NC (a-c) and TV-NC (d-f), respectively.
(JPG)

S3 Fig Fitting of thermodynamic model to adsorption of Cu(II), Ni(II), and Cr(VI) on to TK-NC (a-c) and TV-NC (d-f), respectively.
(JPG)

S4 Fig The zeta potential under different pH and pH_(PZC)_ for TK-NC (a) and TV-NC (b).
(JPG)

S1 Table Comparison of metal ions adsorption potential of various adsorbents with synthesized nanocomposites (TK-NC and TV-NC). (DOCX)

S2 Table FTIR analysis of TK-NC and TV-NC before and after metal adsorption. (DOCX)

S1 File (DOCX)
