# Supplementary figures and images for: Biochar supported metallo-inorganic nanocomposite: A green approach for decontamination of heavy metals from water
Source: PLoS One. 2023 Sep 14;18(9):e0289069. doi: 10.1371/journal.pone.0289069 (PMC10501632; doi:10.1371/journal.pone.0289069)

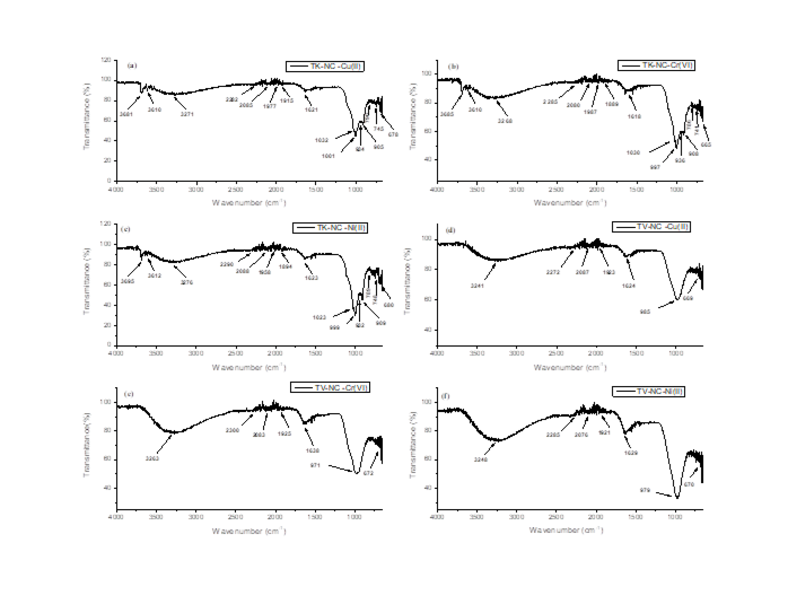

Supplement: S1 Fig — FT-IR of nanocomposites TK-NC (a-c) and TV-NC (d-f) after adsorption of metal ions. (TIF) [file pone.0289069.s001.tif]

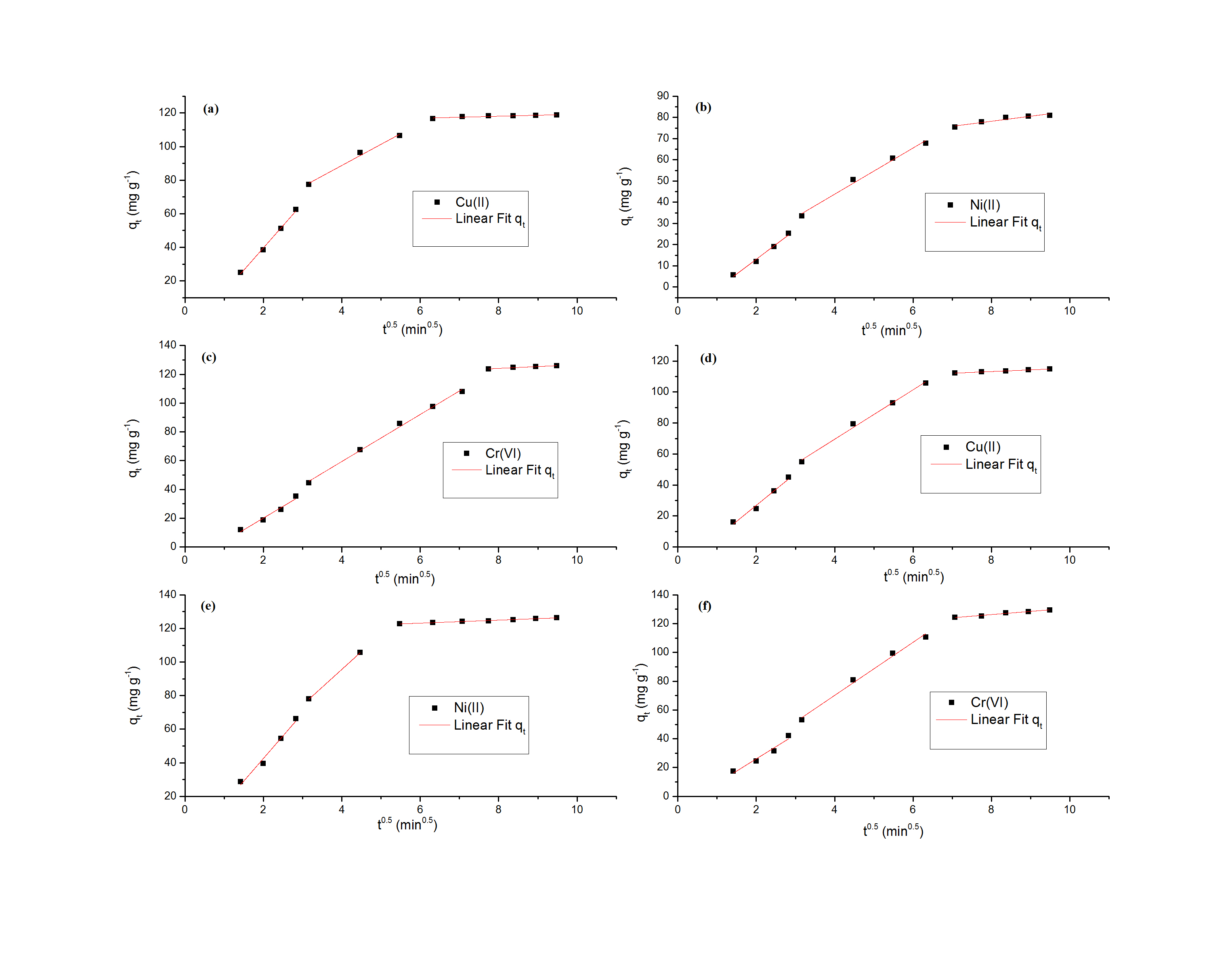

Supplement: S2 Fig — Fitting of intra-particle diffusion model (qt vs t0.5) to adsorption of Cu(II), Ni(II), and Cr(VI) on to TK-NC (a-c) and TV-NC (d-f), respectively. (JPG) [file pone.0289069.s002.jpg]

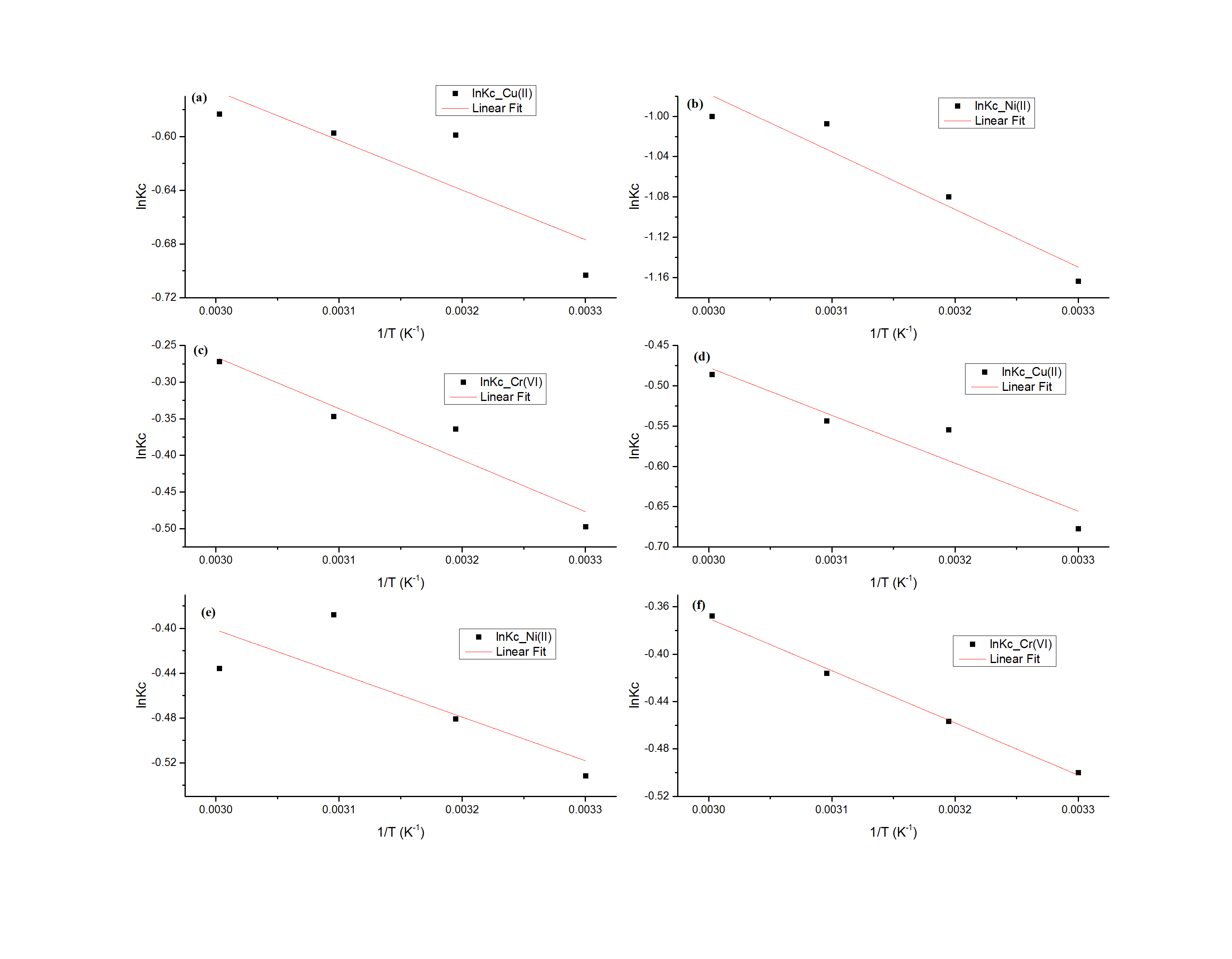

Supplement: S3 Fig — Fitting of thermodynamic model to adsorption of Cu(II), Ni(II), and Cr(VI) on to TK-NC (a-c) and TV-NC (d-f), respectively. (JPG) [file pone.0289069.s003.jpg]

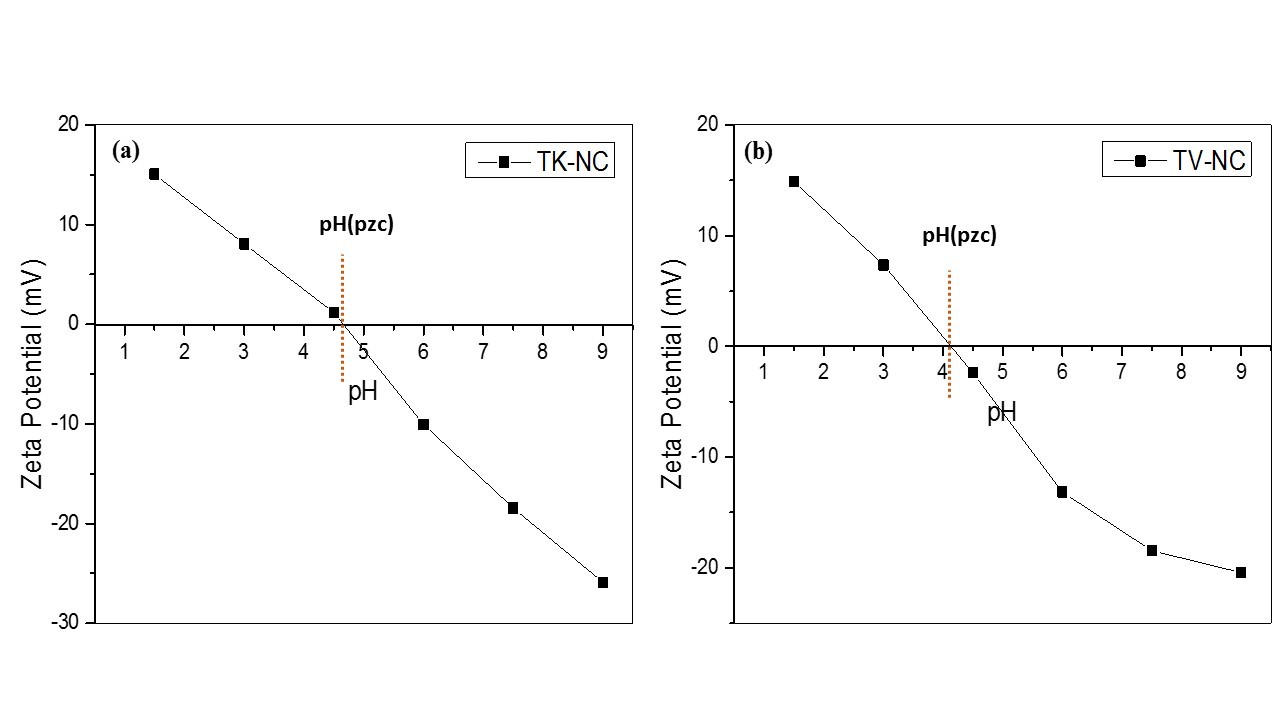

Supplement: S4 Fig — The zeta potential under different pH and pH(PZC) for TK-NC (a) and TV-NC (b). (JPG) [file pone.0289069.s004.jpg]
