# Supplementary material for: Biochar supported metallo-inorganic nanocomposite: A green approach for decontamination of heavy metals from water
Source: PLoS One. 2023 Sep 14;18(9):e0289069. doi: 10.1371/journal.pone.0289069 (PMC10501632; doi:10.1371/journal.pone.0289069)
Supplement: S1 Table — (DOCX) [file pone.0289069.s005.docx]

**S1 Table** Comparison of metal ions adsorption potential of various adsorbents with synthesized nanocomposites (TK-NC and TV-NC)

| Adsorbent | Metal ion removed | Adsorption capacity (mg g^-1^) | Kinetic and isotherm models | Equilibrium time | References |
| --- | --- | --- | --- | --- | --- |
| Iron-zinc ferrate decorated *Macadamia* nutshell biochar | Cr(VI) | 13.90-31.22 | Pseudo-second, Freundlich | 90 mins | [[1](#_ENREF_1)] |
| Fe/Mn oxide loaded corn straw biochar | Cr(VI) | 45.78 | Pseudo-second, Langmuir | 1440 mins | [[2](#_ENREF_2)] |
| Montmorillonite modified peanut shell biochar | Cr(VI) | 12.18 | Pseudo-first-order, Langmuir | 80 mins | [[3](#_ENREF_3)] |
| Acetic acid activated kaolinite  Hydrochloric acid activated kaolinite | Cr(VI) | 10.42  18.15 | Pseudo-second-order, Langmuir | 513 mins  2631 mins | [[4](#_ENREF_4)] |
| Chemically modified chitosan incorporated montmorillonite | Cr(VI) | 15.67 | Pseudo-second-order, Langmuir | 120 mins | [[5](#_ENREF_5)] |
| Spent Tea waste biochar | Cr(VI) | 198.00 | Pseudo-second, Freundlich | 120 min | [[6](#_ENREF_6)] |
| Hydrocalcite/hydroxyapatite impregnated carbon nanotubes | Cr(VI) | 76.97% | Pseudo-second, Freundlich | 600 mins | [[7](#_ENREF_7)] |
| Fe_2_O_3_/SiO_2_ anchored kaolinite | Cu(II) | 153.85 | Langmuir | 60 mins | [[8](#_ENREF_8)] |
| Iron doped Ethiopian nano-clay | Cu(II) | 11.97 | Pseudo-second-order, Langmuir | 150 mins | [[9](#_ENREF_9)] |
| Coal fly ash modified hydroxyapatite | Cu(II) | 44.40-73.60 | Pseudo-second-order,  R-P | 120 mins | [[10](#_ENREF_10)] |
| MnO_2_/Mn_3_O_4_ supported hickory wood biochar | Cu(II) | 34.20 | Pseudo-second, R-P | 480 mins | [[11](#_ENREF_11)] |
| Hydroxyapatite doped *Undaria pinnatifida* roots biochar | Cu(II) | 99.01 | Pseudo-second, Langmuir | 480 mins | [[12](#_ENREF_12)] |
| Magnetic *Zea mays* derived biochar | Cu(II)  Ni(II) | 23.60  22.53 | Pseudo-second-order, Langmuir | 120 mins Cu(II), 90 mins Ni(II) | [[13](#_ENREF_13)] |
| MnFe_2_O_4_ functionalized graphene oxide | Ni(II) | 152.67 | Pseudo-second-order, Langmuir | 270 mins | [[14](#_ENREF_14)] |
| Hydrous zirconium oxide nanoparticles incorporated vermiculite | Ni(II) | 90.21 | Pseudo-second, Langmuir | 120 mins | [[15](#_ENREF_15)] |
| Hydrocalcite/hydroxyapatite impregnated carbon nanotubes | Cr(VI) | 76.97% | Pseudo-second, Freundlich | 600 mins | [[7](#_ENREF_7)] |
| Iron nanoparticles impregnated tea waste | Ni(II) | 200.80 | Pseudo-second, Langmuir | 110 min | [[16](#_ENREF_16)] |
| Magnetic nanoparticles decorated tea waste biochar | Ni(II) | 147.80 | Pseudo-second, Langmuir | 4320 mins | [[17](#_ENREF_17)] |
| Shrimp waste modified *T. Natans* husk biochar | Ni(II) | 44.78 | Pseudo-second, Langmuir | 300 mins | [[18](#_ENREF_18)] |
| MgO modified leafy trash biochar | As(V)  Pb(II) | 157.00  103.00 | Langmuir, pseudo- second order | 48 h | [[19](#_ENREF_19)] |
| Corn stalk biochar loaded with MnO_2_ | Cu(II) | 142.00 | Langmuir, pseudo- second order | 600min | [[20](#_ENREF_20)] |
| MoS_2_ doped saw dust biochar | Pb(II) | 189.00 | Langmuir, pseudo- second order | ~90min | [[21](#_ENREF_21)] |
| Magnetic camel bone biochar | Pb(II)  Co(II)  Cd(II) | 344.80  294.10  322.60 | Langmuir, pseudo- second order | 120 min | [[22](#_ENREF_22)] |
| TK-NC | Cu(II)  Ni(II)  Cr(VI) | 252.65  204.68  343.05 | General order, R-P | 40 mins Cu(II), 50 mins Ni(II), 60 mins Cr(VI) | **This study** |
| TV-NC | Cu(II)  Ni(II)  Cr(VI) | 261.87  230.34  281.82 | General order, R-P | 50 mins (Cu(II), Cr(VI)), 30 mins Ni(II) | **This study** |
