# Supplementary material for: Biochar supported metallo-inorganic nanocomposite: A green approach for decontamination of heavy metals from water
Source: PLoS One. 2023 Sep 14;18(9):e0289069. doi: 10.1371/journal.pone.0289069 (PMC10501632; doi:10.1371/journal.pone.0289069)
Supplement: S2 Table — (DOCX) [file pone.0289069.s006.docx]

**S2** **Table** FTIR analysis of TK-NC and TV-NC before and after metal adsorption

| **Band position before metal adsorption (TK-NC) cm^-1^** | **Band position after metal adsorption (TK-NC) cm^-1^** | | | **Surface functional group assignment** | **Band position before metal adsorption (TV-NC) cm^-1^** | **Band position after metal adsorption (TV-NC) cm^-1^** | | | **Surface functional group assignment** |
| --- | --- | --- | --- | --- | --- | --- | --- | --- | --- |
|  | **Cu(II)** | **Cr(VI)** | **Ni(II)** |  |  | **Cu(II)** | **Cr(VI)** | **Ni(II)** |  |
| 3690 | 3681 | 3685 | 3695 | -OH stretching | 3289 | 3241 | 3263 | 3248 | -OH stretching |
| 3617 | 3610 | 3610 | 3612 | -OH stretching | 2258 | 2272 | 2300 | 2285 | C=C stretching |
| 3285 | 3271 | 3268 | 3276 | -OH stretching | 2095 | 2087 | 2083 | 2076 | C=C stretching |
| 2300 | 2282 | 2285 | 2290 | C≡C stretching | 1937 | 1923 | 1925 | 1921 | C=O vibrations |
| 2095 | 2085 | 2080 | 2088 | C≡C stretching | 1649 | 1624 | 1638 | 1629 | C=C vibrations |
| 1996 | 1977 | 1987 | 1958 | C=O vibrations | 1057 | disappeared | disappeared | disappeared | PO_4_^3-^ asymmetrical vibrations |
| 1876 | 1915 | 1889 | 1894 | C=O vibrations | 1002 | 985 | 971 | 979 | Si-O |
| 1627 | 1621 | 1615 | 1623 | C=C , C=O vibrations | 682 | 669 | 672 | 670 | Mg/Al-OH |
| 1027 | 1032 | 1030 | 1023 | PO_4_^3-^ asymmetrical vibrations | - | - | - | - | - |
| 1009 | 1001 | 997 | 999 | Si-O | - | - | - | - | - |
| 940 | 934 | 936 | 933 | Al-OH | - | - | - | - | - |
| 918 | 905 | 908 | 909 | Al-OH | - | - | - | - | - |
| 796 | 790 | 786 | 789 | Si-O-Al | - | - | - | - | - |
| 750 | 745 | 741 | 748 | Si-O-Al | - | - | - | - | - |
| 670 | 678 | 667 | 680 | Al-OH | - | - | - | - | - |
