# Supplementary material for: Biochar supported metallo-inorganic nanocomposite: A green approach for decontamination of heavy metals from water
Source: PLoS One. 2023 Sep 14;18(9):e0289069. doi: 10.1371/journal.pone.0289069 (PMC10501632; doi:10.1371/journal.pone.0289069)
Supplement: S1 File — (DOCX) [file pone.0289069.s007.docx]

**Fig. S1.** FT-IR of nanocomposites TK-NC (a-c) and TV-NC (d-f) after adsorption of metal ions

**Fig. S2.** Fitting of intra-particle diffusion model (q_t_ vs t^0.5^) to adsorption of Cu(II), Ni(II), and Cr(VI) on to TK-NC (a-c) and TV-NC (d-f), respectively.

**Fig. S3.** Fitting of thermodynamic model to adsorption of Cu(II), Ni(II), and Cr(VI) on to TK-NC (a-c) and TV-NC (d-f), respectively.


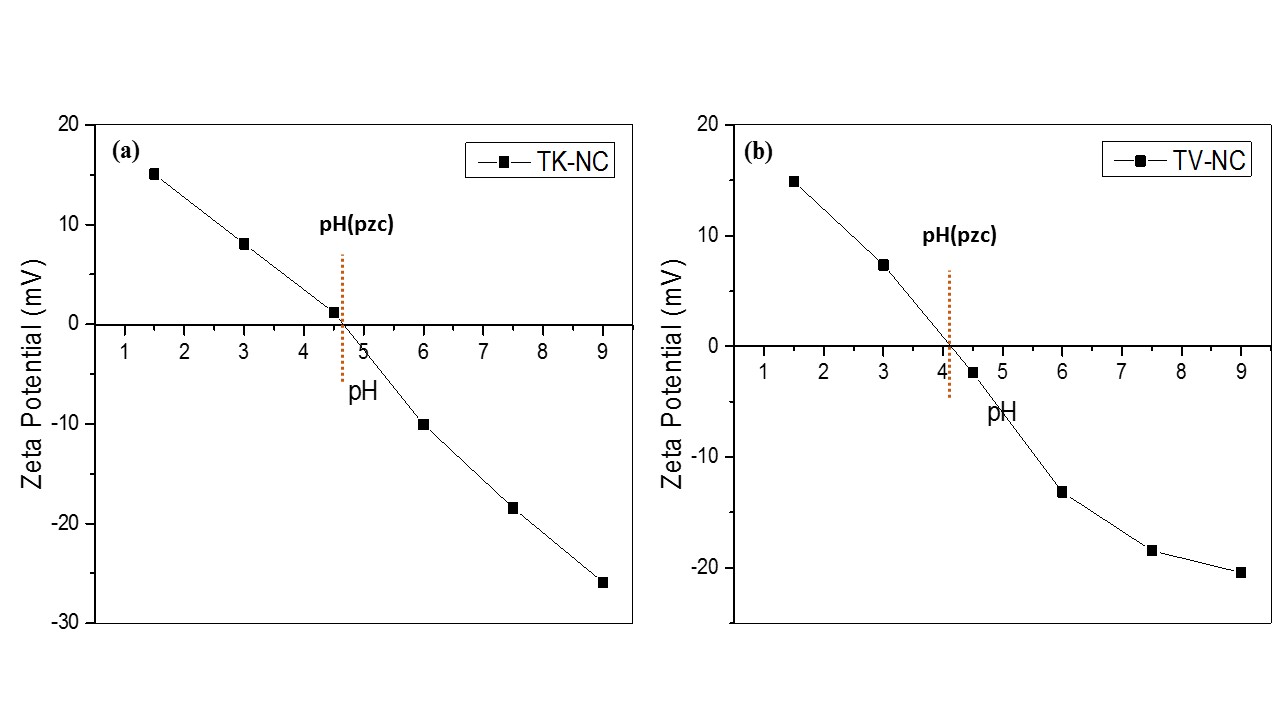


**Fig. S4.** The zeta potential under different pH and pH_(PZC)_ for TK-NC (a) and TV-NC (b)

**Table S1** Comparison of metal ions adsorption potential of various adsorbents with synthesized nanocomposites (TK-NC and TV-NC)

| Adsorbent | Metal ion removed | Adsorption capacity (mg g^-1^) | Kinetic and isotherm models | Equilibrium time | References |
| --- | --- | --- | --- | --- | --- |
| Iron-zinc ferrate decorated *Macadamia* nutshell biochar | Cr(VI) | 13.90-31.22 | Pseudo-second, Freundlich | 90 mins | ([1](#_ENREF_1)) |
| Fe/Mn oxide loaded corn straw biochar | Cr(VI) | 45.78 | Pseudo-second, Langmuir | 1440 mins | ([2](#_ENREF_2)) |
| Montmorillonite modified peanut shell biochar | Cr(VI) | 12.18 | Pseudo-first-order, Langmuir | 80 mins | ([3](#_ENREF_3)) |
| Acetic acid activated kaolinite  Hydrochloric acid activated kaolinite | Cr(VI) | 10.42  18.15 | Pseudo-second-order, Langmuir | 513 mins  2631 mins | ([4](#_ENREF_4)) |
| Chemically modified chitosan incorporated montmorillonite | Cr(VI) | 15.67 | Pseudo-second-order, Langmuir | 120 mins | ([5](#_ENREF_5)) |
| Spent Tea waste biochar | Cr(VI) | 198.00 | Pseudo-second, Freundlich | 120 min | ([6](#_ENREF_6)) |
| Hydrocalcite/hydroxyapatite impregnated carbon nanotubes | Cr(VI) | 76.97% | Pseudo-second, Freundlich | 600 mins | ([7](#_ENREF_7)) |
| Fe_2_O_3_/SiO_2_ anchored kaolinite | Cu(II) | 153.85 | Langmuir | 60 mins | ([8](#_ENREF_8)) |
| Iron doped Ethiopian nano-clay | Cu(II) | 11.97 | Pseudo-second-order, Langmuir | 150 mins | ([9](#_ENREF_9)) |
| Coal fly ash modified hydroxyapatite | Cu(II) | 44.40-73.60 | Pseudo-second-order,  R-P | 120 mins | ([10](#_ENREF_10)) |
| MnO_2_/Mn_3_O_4_ supported hickory wood biochar | Cu(II) | 34.20 | Pseudo-second, R-P | 480 mins | ([11](#_ENREF_11)) |
| Hydroxyapatite doped *Undaria pinnatifida* roots biochar | Cu(II) | 99.01 | Pseudo-second, Langmuir | 480 mins | ([12](#_ENREF_12)) |
| Magnetic *Zea mays* derived biochar | Cu(II)  Ni(II) | 23.60  22.53 | Pseudo-second-order, Langmuir | 120 mins Cu(II), 90 mins Ni(II) | ([13](#_ENREF_13)) |
| MnFe_2_O_4_ functionalized graphene oxide | Ni(II) | 152.67 | Pseudo-second-order, Langmuir | 270 mins | ([14](#_ENREF_14)) |
| Hydrous zirconium oxide nanoparticles incorporated vermiculite | Ni(II) | 90.21 | Pseudo-second, Langmuir | 120 mins | ([15](#_ENREF_15)) |
| Hydrocalcite/hydroxyapatite impregnated carbon nanotubes | Cr(VI) | 76.97% | Pseudo-second, Freundlich | 600 mins | ([7](#_ENREF_7)) |
| Iron nanoparticles impregnated tea waste | Ni(II) | 200.80 | Pseudo-second, Langmuir | 110 min | ([16](#_ENREF_16)) |
| Magnetic nanoparticles decorated tea waste biochar | Ni(II) | 147.80 | Pseudo-second, Langmuir | 4320 mins | ([17](#_ENREF_17)) |
| Shrimp waste modified *T. Natans* husk biochar | Ni(II) | 44.78 | Pseudo-second, Langmuir | 300 mins | ([18](#_ENREF_18)) |
| MgO modified leafy trash biochar | As(V)  Pb(II) | 157.00  103.00 | Langmuir, pseudo- second order | 48 h | ([19](#_ENREF_19)) |
| Corn stalk biochar loaded with MnO_2_ | Cu(II) | 142.00 | Langmuir, pseudo- second order | 600min | ([20](#_ENREF_20)) |
| MoS_2_ doped saw dust biochar | Pb(II) | 189.00 | Langmuir, pseudo- second order | ~90min | ([21](#_ENREF_21)) |
| Magnetic camel bone biochar | Pb(II)  Co(II)  Cd(II) | 344.80  294.10  322.60 | Langmuir, pseudo- second order | 120 min | ([22](#_ENREF_22)) |
| TK-NC | Cu(II)  Ni(II)  Cr(VI) | 252.65  204.68  343.05 | General order, R-P | 40 mins Cu(II), 50 mins Ni(II), 60 mins Cr(VI) | **This study** |
| TV-NC | Cu(II)  Ni(II)  Cr(VI) | 261.87  230.34  281.82 | General order, R-P | 50 mins (Cu(II), Cr(VI)), 30 mins Ni(II) | **This study** |

**Table S2** FTIR analysis of TK-NC and TV-NC before and after metal adsorption

| **Band position before metal adsorption (TK-NC) cm^-1^** | **Band position after metal adsorption (TK-NC) cm^-1^** | | | **Surface functional group assignment** | **Band position before metal adsorption (TV-NC) cm^-1^** | **Band position after metal adsorption (TV-NC) cm^-1^** | | | **Surface functional group assignment** |
| --- | --- | --- | --- | --- | --- | --- | --- | --- | --- |
|  | **Cu(II)** | **Cr(VI)** | **Ni(II)** |  |  | **Cu(II)** | **Cr(VI)** | **Ni(II)** |  |
| 3690 | 3681 | 3685 | 3695 | -OH stretching | 3289 | 3241 | 3263 | 3248 | -OH stretching |
| 3617 | 3610 | 3610 | 3612 | -OH stretching | 2258 | 2272 | 2300 | 2285 | C=C stretching |
| 3285 | 3271 | 3268 | 3276 | -OH stretching | 2095 | 2087 | 2083 | 2076 | C=C stretching |
| 2300 | 2282 | 2285 | 2290 | C≡C stretching | 1937 | 1923 | 1925 | 1921 | C=O vibrations |
| 2095 | 2085 | 2080 | 2088 | C≡C stretching | 1649 | 1624 | 1638 | 1629 | C=C vibrations |
| 1996 | 1977 | 1987 | 1958 | C=O vibrations | 1057 | disappeared | disappeared | disappeared | PO_4_^3-^ asymmetrical vibrations |
| 1876 | 1915 | 1889 | 1894 | C=O vibrations | 1002 | 985 | 971 | 979 | Si-O |
| 1627 | 1621 | 1615 | 1623 | C=C , C=O vibrations | 682 | 669 | 672 | 670 | Mg/Al-OH |
| 1027 | 1032 | 1030 | 1023 | PO_4_^3-^ asymmetrical vibrations | - | - | - | - | - |
| 1009 | 1001 | 997 | 999 | Si-O | - | - | - | - | - |
| 940 | 934 | 936 | 933 | Al-OH | - | - | - | - | - |
| 918 | 905 | 908 | 909 | Al-OH | - | - | - | - | - |
| 796 | 790 | 786 | 789 | Si-O-Al | - | - | - | - | - |
| 750 | 745 | 741 | 748 | Si-O-Al | - | - | - | - | - |
| 670 | 678 | 667 | 680 | Al-OH | - | - | - | - | - |

**References**

1. Qhubu MC, Methula B, Xaba T, Moyo M, Pakade VE. Iron-Zinc Impregnated Biochar Composite as a Promising Adsorbent for Toxic Hexavalent Chromium Remediation: Kinetics, Isotherms and Thermodynamics. Chem Afr. 2021:1-11.

2. Zhu Y, Dai W, Deng K, Pan T, Guan Z. Efficient removal of Cr (VI) from aqueous solution by Fe-Mn oxide-modified biochar. Water Air Soil Pollut. 2020;231:1-17.

3. Wang H, Tan L, Hu B, Qiu M, Liang L, Bao L, et al. Removal of Cr (VI) from acid mine drainage with clay-biochar composite. Environment. 2019;28:32.

4. Dim P, Mustapha L, Termtanun M, Okafor J. Adsorption of chromium (VI) and iron (III) ions onto acid-modified kaolinite: Isotherm, kinetics and thermodynamics studies. Arab J Chem. 2021;14(4):103064.

5. Wang Y-M, Duan L, Sun Y, Hu N, Gao J-Y, Wang H, et al. Adsorptive removal of Cr (VI) from aqueous solutions with an effective adsorbent: cross-linked chitosan/montmorillonite nanocomposites in the presence of hydroxy-aluminum oligomeric cations. Desalin WaterTreat. 2016;57(23):10767-75.

6. Khalil U, Shakoor MB, Ali S, Rizwan M, Alyemeni MN, Wijaya L. Adsorption-reduction performance of tea waste and rice husk biochars for Cr (VI) elimination from wastewater. J Saudi Chem Soc. 2020;24(11):799-810.

7. Rodrigues E, Almeida O, Brasil H, Moraes D, dos Reis M. Adsorption of chromium (VI) on hydrotalcite-hydroxyapatite material doped with carbon nanotubes: Equilibrium, kinetic and thermodynamic study. Appl Clay Sci. 2019;172:57-64.

8. Awwad AM, Amer MA. Adsorption of Pb (II), Cd (II), and Cu (II) ions onto SiO2/kaolinite/Fe2O3 composites: modeling and thermodynamics properties. Chem Int. 2022;8(3):95-100.

9. Tarekegn MM, Balakrishnan RM, Hiruy AM, Dekebo AH, Maanyam HS. Nano-Clay and Iron Impregnated Clay Nanocomposite for Cu2+ and Pb2+ Ions Removal from Aqueous Solutions. Air Soil Water Res. 2022;15:11786221221094037.

10. Sočo E, Papciak D, Michel MM, Pająk D, Domoń A, Kupiec B. Characterization of the Physical, Chemical, and Adsorption Properties of Coal-Fly-Ash–Hydroxyapatite Composites. Minerals. 2021;11(7):774.

11. Wang H, Gao B, Wang S, Fang J, Xue Y, Yang K. Removal of Pb (II), Cu (II), and Cd (II) from aqueous solutions by biochar derived from KMnO4 treated hickory wood. Bioresour Technol. 2015;197:356-62.

12. Deng Y, Li X, Ni F, Liu Q, Yang Y, Wang M, et al. Synthesis of magnesium modified biochar for removing copper, lead and cadmium in single and binary systems from aqueous solutions: adsorption mechanism. Water. 2021;13(5):599.

13. Isaac R, Siddiqui S. Sequestration of Ni (II) and Cu (II) using FeSO4 modified Zea mays husk magnetic biochar: Isotherm, kinetics, thermodynamic studies and RSM. J Hazard Mater Adv. 2022;8:100162.

14. Thy LTM, Kiem NH, Tu TH, Phu LM, Oanh DTY, Nam HM, et al. Fabrication of manganese ferrite/graphene oxide nanocomposites for removal of nickel ions, methylene blue from water. Chem Phys. 2020;533:110700.

15. Liu D, Deng S, Vakili M, Du R, Tao L, Sun J, et al. Fast and high adsorption of Ni (II) on vermiculite-based nanoscale hydrated zirconium oxides. Chem Eng J. 2019;360:1150-7.

16. Butt RS, Nazir R, Khan MN, Hamid A, Deeba F. Treatment of electroplating industry wastewater using iron nanoparticle doped spent tea waste charcoal. J Biodivers Environ Sci. 2014;5:7-17.

17. Shirvanimoghaddam K, Czech B, Tyszczuk-Rotko K, Kończak M, Fakhrhoseini SM, Yadav R, et al. Sustainable synthesis of rose flower-like magnetic biochar from tea waste for environmental applications. J Adv Res. 2021;34:13-27.

18. Yin W, Zhang W, Zhao C, Xu J. Evaluation of removal efficiency of Ni (II) and 2, 4-DCP using in situ nitrogen-doped biochar modified with aquatic animal waste. ACS Omega. 2019;4(21):19366-74.

19. Li R, Liang W, Wang JJ, Gaston LA, Huang D, Huang H, et al. Facilitative capture of As (V), Pb (II) and methylene blue from aqueous solutions with MgO hybrid sponge-like carbonaceous composite derived from sugarcane leafy trash. J Environ Manage. 2018;212:77-87.

20. Zhou L, Huang Y, Qiu W, Sun Z, Liu Z, Song Z. Adsorption properties of nano-MnO2–biochar composites for copper in aqueous solution. Molecules. 2017;22(1):173.

21. Zhu H, Tan X, Tan L, Chen C, Alharbi NS, Hayat T, et al. Biochar derived from sawdust embedded with molybdenum disulfide for highly selective removal of Pb2+. ACS Applied Nano Materials. 2018;1(6):2689-98.

22. Alqadami AA, Khan MA, Otero M, Siddiqui MR, Jeon B-H, Batoo KM. A magnetic nanocomposite produced from camel bones for an efficient adsorption of toxic metals from water. J Clean Prod. 2018;178:293-304.
